# Extraintestinal traits of pathogenicity and sequence type lineages in commensal *Escherichia coli* from adults and young children: genotypic and phenotypic profiles

**DOI:** 10.3389/fmicb.2025.1579685

**Published:** 2025-05-26

**Authors:** Ewa Bok, Justyna Mazurek-Popczyk, Magdalena Wojciech, Katarzyna Baldy-Chudzik

**Affiliations:** ^1^Department of Microbiology and Molecular Biology, Institute of Health Sciences, Collegium Medicum, University of Zielona Góra, Zielona Góra, Poland; ^2^Department of Applied Mathematics, Institute of Mathematics, Faculty of Exact and Natural Sciences, University of Zielona Góra, Zielona Góra, Poland

**Keywords:** commensal *Escherichia coli*, pathogenicity-associated islands, genotypic background, phenotypic traits, sequence type, mathematical optimization, multiple testing

## Abstract

**Background:**

The commensal *Escherichia coli* population may significantly influence the pathogenesis of extraintestinal infections. The assignment to specific sequence type (ST) lineages and the presence of particular combinations of virulence genes are characteristic features of extraintestinal pathogenic *E. coli* (ExPEC)—although not exclusively. Extraintestinal virulence genes are also identified among commensal *E. coli*. This study aimed to examine the genotypic and phenotypic characteristics of the extraintestinal virulence potential of two populations of commensal *E. coli* isolates from adults and young children.

**Methods:**

Genotypic traits were detected using polymerase chain reaction (PCR). Appropriate phenotypic assays and real-time PCR were used to analyze the expression of virulence factors. Multilocus sequence typing was performed using the seven-loci Achtman scheme.

**Results:**

Genotypic studies revealed the virulence potential of the commensal isolates, and phenotypic analyses confirmed whether the genes are expressed. *E. coli* from adults carried pathogenicity islands and virulence genes in significantly higher proportions, resulting from the dominance of phylogroup B2 in this set of isolates. The hemolytic activity and higher levels of siderophore receptor expression were more common in *E. coli* from adults and were closely related to the dominance of phylogroup B2. Other traits not associated with phylogroup B2, such as adhesion abilities mediated by type 1 and P fimbriae and strong biofilm formation propensities, were detected with similar frequencies in both pools of isolates. *E. coli* harboring pathogenicity islands were subjected to multilocus sequence typing analysis. Sequence types ST73, ST59, ST131, ST95, ST141, and ST69 were most common in isolates from adults, whereas ST10, ST155, ST59, and ST1823 from children. In our collection of *E. coli*, the ST73 exhibited the highest potential for extraintestinal virulence.

**Conclusion:**

A significant proportion of *E. coli* from adults compared to young children exhibited considerable virulence potential, which may enable them to function as endogenous pathogens. Our research highlights the significant accumulation of extraintestinal pathogenicity features in commensal *E. coli*, indicating the need to monitor genetic and phenotypic profiles in “silent” potential pathogens.

## Introduction

*Escherichia coli* is one of the most genetically diverse microorganisms, capable of colonizing and thriving in various vertebrate host niches or environmental habitats (Braz et al., 2020; Denamur et al., 2021). In humans, it is a member of the physiological gut microbiota (Tenaillon et al., 2010; Martinson and Walk, 2020). However, some commensal *E. coli* can cause infections outside the gastrointestinal tract, such as urinary tract infections (UTIs), sepsis, neonatal meningitis, pulmonary infections, and constitute a rather specific group of opportunistic pathogens (Dobrindt et al., 2013; Frankel and Ron, 2018; Pokharel et al., 2023). Such *E. coli* strains are called Extraintestinal pathogenic *E. coli* (ExPEC) (Kaper et al., 2004; Dale and Woodford, 2015). Global morbidity and mortality rates due to ExPEC infections are significant and increasing over time, leading to economic losses for health systems (Poolman and Wacker, 2016; Frankel and Ron, 2018). The urinary tract is the most common extraintestinal site infected by *E. coli*, and consequently, a common source of bloodstream infections (Russo and Johnson, 2003; Paramita et al., 2020). ExPEC strains isolated from clinical cases show significant differences in genome size, phylogenetic affiliation, and serotypes (Moreno et al., 2008; McLellan and Hunstad, 2016). This significant diversity results from the plastic structure of the genome, which is influenced by horizontal gene transfer (HGT) or homologous recombination, enabling bacteria to adapt to new environments (Dobrindt et al., 2003, 2013). The characteristic feature of ExPEC is the carriage of numerous specific virulence genes (VGs) encoding adhesins, iron acquisition systems, protectins, and toxins on larger genetic platforms termed pathogenicity islands (PAIs) (Östblom et al., 2011; Dobrindt et al., 2013; Sarowska et al., 2019; Denamur et al., 2021). Clinical ExPEC strains are often defined by their number and constellation of VGs; however, a precise characterization is not possible because they lack a distinct and unique set of VGs specific to a particular disease type. Various combinations of VGs can lead to the same extraintestinal disease outcome, which defines an ExPEC pathotype (Köhler and Dobrindt, 2011; Dobrindt et al., 2013; Manges et al., 2019).

Numerous reports mention the presence of ExPEC virulence genes in commensal *E. coli* (Nowrouzian et al., 2003; Vollmerhausen et al., 2011; Starčič Erjavec and Žgur-Bertok, 2015; Bok et al., 2018). These strains commonly harbor genes that encode P fimbriae, type 1 fimbriae, capsular antigens (K1 and K5), *α*-hemolysin, and aerobactin, and often belong to phylogroups B2 and D, which are associated with extraintestinal pathogenicity. It was hypothesized that these factors might primarily be related to fitness, with extraintestinal pathogenicity serving as a secondary effect. Regarding the prevalence of these virulence or fitness-associated genes and their phylogroup classification, these strains resemble typical ExPEC isolates (Nowrouzian et al., 2001b, 2001a; Dobrindt et al., 2013). It was also reported that commensal *E. coli* often carry PAIs associated with the aforementioned VGs. This suggests that the intestinal microbiota may act as a reservoir for bacteria that can cause extraintestinal infections (Sabaté et al., 2006; Li et al., 2010). Such strains can exist asymptomatically for a long time in the intestines, making the line between commensalism and pathogenicity very thin and rendering it nearly impossible to distinguish commensals from these pathogens (Tenaillon et al., 2010; Köhler and Dobrindt, 2011; Dobrindt et al., 2013).

The presence of virulence genes among *E. coli* strains in the colonic microbiota has been confirmed in many studies (Nowrouzian et al., 2003; Gordon et al., 2005; Moreno et al., 2009; Vollmerhausen et al., 2011; Al Mayahie, 2014; Starčič Erjavec and Žgur-Bertok, 2015; Bok et al., 2018). Nevertheless, the extent to which these genes are expressed and may reveal virulence characteristics remains uncertain. There are a limited studies concerning phenotypic analysis of ExPEC-related virulence factors. Commensal *E. coli* strains have been reported to exhibit adhesion to the colonic cell line HT-29 (Nowrouzian et al., 2006). Another comparative study showed that fecal *E. coli* display higher mannose-sensitive (MS) hemagglutination but lower mannose-resistant (MR) hemagglutination and hemolysis than pathogenic strains (Shruthi, 2012). The analysis of biofilm formation indicated a strong ability to form biofilm in 33% of commensal *E. coli* strains (Raimondi et al., 2019). Some reports indicated that fecal and rectal *E. coli* strains could produce siderophores associated with extraintestinal virulence (Henderson et al., 2009; Searle et al., 2015).

Multilocus sequence typing (MLST) is becoming increasingly important in characterizing ExPEC strains to identify clonal complexes gene lineages (Riley, 2014; Manges et al., 2019). The analyses of ExPEC associated with both community-onset and healthcare-associated infections, particularly urinary tract infections and bloodstream infections, from various continents have demonstrated that lineages including sequence types (STs): ST131, ST95, ST73, ST69, ST393, ST10, ST12, ST38, and ST405, are globally disseminated and responsible for the majority of ExPEC infections (Riley, 2014; Manges et al., 2019; Denamur et al., 2021; Manges et al., 2019). A limited number of studies focused on *E. coli* stool isolates, which may explain why many STs had low detection rates (Massot et al., 2016; Manges et al., 2019; Marin et al., 2022).

Considering that ExPEC strains originate from intestinal *E. coli* and can exist asymptomatically in the human intestine for years as commensals, it is unlikely that these strains can be eradicated. Hence, their monitoring is critical to obtain the widest possible knowledge about the spread of such strains in different populations of healthy individuals. In this study, a collection of fecal *E. coli* from healthy adults and young children was characterized to estimate the proportion of strains exhibiting ExPEC features. We examined the extended phylogenetic structure, presence of PAIs, VGs, the structure of the two fimbrial operons for type I and P fimbriae, and the ability to undergo MS and MR agglutination, hemolysis, biofilm formation, and expression of siderophore receptors. In addition, MLST was performed, and STs were determined.

## Materials and methods

### Sample collection, *E. coli* identification, and study design

In this study, we analyze the set of 143 commensal *E. coli* isolates from adults aged 18–54 years and 50 from young children aged 0.5–3 years. These isolates complement the broader collection described in detail in a previous study (Bok et al., 2018). Briefly, fecal samples from adults and young children were collected in 2018–2020. Each sample was represented by a single colony showing a typical *E. coli* morphology on MacConkey agar, randomly selected and identified using MicroScan AS4 (Beckman Coulter). In doubtful cases, the species *E. coli* was additionally confirmed by the BD™ Bruker MALDI Biotyper. *E. coli* isolates were frozen as a glycerol stock at −80°C until further analysis. The DNA extraction was carried out using the thermal cell lysis method; 3–6 μL of the boiled bacterial supernatant was used as a template in all the PCR reactions.

The experimental design is presented in the supplementary material (Supplementary Figure S1).

### Extended phylogenetic typing

The phylogenetic designations of the *E. coli* isolates were determined by following the revised phylogenetic typing method described by Clermont et al. in 2013. *E. coli* isolates were classified into phylogroups A, B1, B2, D, and F using a new quadruplex PCR. Additional specific PCR screening was performed to assign *E. coli* into phylogroups C, E, and clade I (Clermont et al., 2011, 2013). *E. coli* reference (ECOR) collection strains (Institut Pasteur Collection, Paris, France) were examined as positive and negative controls, respectively, in all PCR reactions.

### Pathogenicity islands, virulence genes, and fimbrial operons genotyping

The *E. coli* isolates were screened for the presence of the five most prevalent PAIs typical for uropathogenic *E. coli*: PAI I CFT073, PAI II CFT073, PAI I 536, PAI IV 536, and PAI II J96. The virulence determinants encoded on these different PAIs are listed as follows: PAI I CFT073—*α*-hemolysin, P-fimbriae and aerobactin; PAI II CFT073—P-fimbriae and iron-regulated genes; PAI I 536—α-hemolysin, CS12 fimbriae, and F17-like fimbrial adhesin; PAI IV 536—yersiniabactin siderophore system; PAI II J96—α-hemolysin, Prs-fimbriae, and cytotoxic necrotising factor 1 (Koga et al., 2014). The *E. coli* isolates were examined also for the presence of the following extraintestinal VGs, representing five functional categories: (1) adhesins: the genes essential for expression of two fimbrial operons, type 1 fimbriae: *fimB*, *fimE*, *fimA*, *fimI*, *fimC*, *fimH*, and *fim* switch region; P fimbriae: *papAH*, *papC*, *papEF*, and *papG* and its variants (*GI*, *GII*, and *GIII*) and *sfaS* (S fimbriae); (2) biofilm formation:, *agn43a*, *agn43b*, and *agn43K12* (antigen 43 alleles a, b, and K12); (3) iron acquisition: *fyuA* (yersiniabactin siderophore receptor), *iutA* (aerobactin siderophore receptor), *iroN* (salmochelin siderophore receptor), *ireA* (iron-regulated element, siderophore receptor); (4) protectins: *kpsMTII* K1, *kpsMTII* K2, *kpsMTII* K5 (group II capsule with K1, K2, and K5 variants), *kpsMTIII* (group III capsule), *ompT* (outer membrane protein, protease), *traT* (serum resistance-associated outer membrane protein), and *iss* (increased serum survival); and (5) toxins: *cnf1* (cytotoxic necrotizing factor 1) and *hlyA* (α-hemolysin). Multiplex or simplex PCR-based genotyping was performed with primers and conditions previously described (Johnson and Stell, 2000; Janben et al., 2001; Schwan et al., 2002; Landraud et al., 2003; Johnson and O’Bryan, 2004; Blumer et al., 2005; Chapman et al., 2006; Sabaté et al., 2006; Nowrouzian et al., 2007; Restieri et al., 2007; Hernandes et al., 2011). The PCR amplification mixture in a volume of 25 μL contained buffer solution (Thermo Scientific, Waltham, MA, USA), 2.5-mM MgCl_2_ (Promega, Madison, WI, USA), 0.5 mM of each deoxynucleotide triphosphates (dNTP) (Promega, Madison, WI, USA), 0.2 μM of each primer (Genomed, Warszawa, Poland), 1 U of Dream Taq Green DNA Polymerase (Thermo Scientific, Waltham, MA, USA), and 3 μL of DNA template. Uropathogenic *E. coli* strain CFT073 American Type Culture Collection (ATCC) 700,928 (Argenta, Poznań, Poland), *E. coli* strains from the ECOR collection (Institut Pasteur Collection, Paris, France) and from our collection of human fecal strains, known to possess (validated by sequencing) or lack the genes of interest, were examined in all PCR reactions as positive and negative controls, respectively. The PCR products were separated in a 1.5 or 2% agarose gel electrophoresis and stained with ethidium bromide.

### Determination of the type 1 fimbria phase switch orientation (ON/OFF)

The orientation (ON or OFF) of the invertible fim switch was determined by PCR amplification of a 559 bp region containing the switch. The PCR products were then cleaved by the endonuclease *Hinf*I, which cuts the invertible DNA element asymmetrically, resulting in different-sized fragments depending on whether bacteria had the switch in the ON or OFF orientation. When the switch element is in the “ON” orientation, the promoter is active and the fimbrial genes are transcribed; when the element is in the “OFF” orientation, the promoter is inactive and the fimbrial genes are not transcribed. Bacterial culture conditions were described previously (Nowrouzian et al., 2007). The PCR products were digested by *Hinf*I (Thermo Fisher Scientific, Waltham, MA, USA) according to the manufacturer’s instructions, separated electrophoretically on a 2% agarose gel, and stained with ethidium bromide.

### Agglutination of yeast cells

The binding activity of FimH was evaluated by a mannose-sensitive yeast agglutination (MSYA) assay according to the method described in a previous study (Scribano et al., 2020) with some modifications. The *E. coli* isolates positive for all the tested type 1 fimbria operon genes and with the fim switch region in the ON orientation were tested. The *E. coli* isolates were grown in the Luria-Bertani (LB) medium for 48 h at 37°C in static conditions to enhance the expression of type 1 fimbriae. Yeasts were grown in YPG broth for 72 h at 25°C in static conditions. An aliquot of the bacterial cultures was washed twice with phosphate-buffered saline (PBS). Bacteria were resuspended in PBS or PBS with 3% D-mannose (Sigma, St. Louis, MO, USA) to an optical density of a sample measured at a wavelength of 600 nanometers (OD600) of approximately 1 and incubated for 2 h to bind mannose. *S. cerevisiae* cells were resuspended in PBS to an OD600 of approximately 4. On a standard microscope slide, 50 μL of the *E. coli* suspension in the absence or presence of D-mannose was mixed with 50 μL of the yeast cell suspension, and incubated for 1 h at room temperature. After incubation, each suspension was gently rocked until the agglutination was assessed visually. The yeast agglutination was determined to be mannose-sensitive when D-mannose inhibited it in a final concentration of 1.5%. All assays were conducted in triplicate. The *E. coli* ATCC strain (type 1 fimbriae positive) was used as a positive control.

### Hemagglutination assay

The determination of the presence of P fimbriae was based on the ability of the strains to agglutinate the sheep erythrocytes by the mannose-resistant hemagglutination assay (MRHA) according to the method described previously (Shah et al., 2019) with some modifications. The *E. coli* isolates positive for all the tested P fimbriae operon genes were examined. The bacteria were grown on BHI agar at 37° C for 24 h, and these growth conditions provided maximum expression of MR fimbriae. Bacteria obtained by scraping growth from a solid culture with a loop were suspended in PBS or PBS with 3% D-mannose (Sigma) to an OD600 of approximately 1 and incubated for 2 h to bind mannose. 50 μL of the bacterial suspension in the absence or presence of D-mannose was mixed with 50 μL of the 5% suspension of the sheep erythrocytes sensitized by tannic acid in PBS. The assays were performed on a microscope slide for 1 h. After incubation, each suspension was gently rocked until the hemagglutination was visually assessed. The hemagglutination was considered mannose-resistant when it occurred in the presence of D-mannose. All assays were conducted in triplicate. The *E. coli* ATCC strain (P fimbriae positive) was used as a positive control.

### Biofilm formation analysis

Biofilm formation was evaluated by a crystal violet assay according to the method described in a previous study (Hou et al., 2014) with some modifications. Overnight cultures in LB medium were diluted in fresh M9 medium to an OD600 of 0.12. 200 μL of diluted cultures were placed in a flat-bottomed 96-well polystyrene microtiter plate (Nunc, Denmark) and incubated for 48 h at 37°C without shaking. After incubation, the OD of the cultures was measured at 630 nm. The medium was removed, and the wells were gently washed twice with PBS to remove unbound cells. The wells were dried for 20 min at 42°C. The adhered bacteria were stained with 1% crystal violet for 15 min. Crystal violet was removed, and the wells were washed twice with PBS to eliminate unbound dye. The plates were air-dried. The dye incorporated by the adhered cells was solubilized in 200 μL of 95% ethanol. After 5 min at room temperature, the OD of the stained attached bacteria and control wells was read at 570 nm. The tests were carried out in quadruplicate. *E. coli* ATCC 25922, previously characterized as a strong biofilm producer, was used as a control strain in all experiments (Naves et al., 2008). The extent of biofilm formation was determined by using the formula SBF = (AB − CW)/G, in which SBF is the specific biofilm formation, AB is the OD570 nm of the attached and stained bacteria, CW is the OD570 nm of the stained control wells containing only bacteria-free medium (to eliminate unspecific or abiotic OD values), and G is the OD630 nm of cells growth in broth. The SBF value obtained for the control strain was assumed to be 100%, and the values for the individual tested strains were compared to this value. The degree of biofilm production was classified into three categories: strong—SBF ≥ 100% of the positive control strain value; moderate—100% > SBF ≥ 50%; and weak—SBF < 50%.

### Hemolytic activity evaluation

Hemolytic activity was detected on Columbia Agar with 5% Sheep Blood (Graso Biotech, Jabłowo, Poland), according to the method previously described (Murase et al., 2012). The *E. coli* isolates positive for the *hlyA* gene were tested. Bacterial cells were grown aerobically in LB medium overnight with shaking at 140 rpm and then were collected by centrifugation, washed once with fresh LB medium, and resuspended in the same medium to an OD600 of approximately 0.8. One microlitre of the bacterial suspension was spotted on Columbia Agar plates. After incubation at 37°C for 24 h, the size of the hemolytic zone, defined as the distance from the colony’s border to the end of the hemolytic zone, produced by each strain was measured. The hemolytic activity was graded as follows: -, no hemolysis; +, less than 1 mm; ++, 1–5 mm; and +++, more than 5 mm. Tests were conducted twice. The *E. coli* ATCC 35,218 strain (with hemolytic activity) was used as a positive control.

### RNA extraction

For the expression analysis, isolates carrying three or four siderophore receptor genes *fyuA*, *iutA*, *iroN*, and *ireA* were selected. LB medium (BTL, Łódź, Poland) overnight cultures were diluted 100-fold in 25 mL of fresh M63 minimal medium and cultured at 37°C with agitation at 140 rpm until the OD600 was approximately 0.9. The RNA was purified using the Promega SV total RNA purification kit (Promega, Madison, WI, USA) according to the manufacturer’s instructions. The RNA was quantified by measuring the 260 nm absorbance on a NanoPhotometer N60 (Implen, Munich, Germany). According to the manufacturer’s instructions, all RNA samples were digested with DNase I (Roche, Basel, Switzerland) to eliminate contaminating genomic DNA.

### cDNA synthesis and real-time PCR

To determine the expression level of four siderophore receptor genes, *fyuA*, *iutA*, *iroN*, and *ireA*, reverse transcription was performed using the Maxima First Strand cDNA Synthesis Kit (Thermo Scientific,Waltham, MA, USA) according to the manufacturer’s protocol. Reaction mixture in a volume of 20 μL contained: 0.5 μg of DNase-treated RNA, 5 × Reaction Mix (reaction buffer, dNTPs, oligo (dT)_18_, and random hexamer primers), Maxima Enzyme Mix (Maxima Reverse Transcriptase and Thermo Scientific RiboLock RNase Inhibitor) (Thermo Scientific, Waltham, MA, USA), RNase-free water. A no-reverse transcriptase control (−RT) was prepared for each DNase-treated RNA sample. Real-Time PCR was performed using Maxima SYBR Green qPCR Master Mix (Thermo Scientific, Waltham, MA, USA) 2× Master Mix (PCR buffer, Maxima Hot Start Taq DNA polymerase, dNTPs, SYBR Green I dye) following the manufacturer’s instructions. The reaction mixture in a volume of 25 μL contained 2 × Master Mix (PCR buffer, Maxima Hot Start Taq DNA polymerase, dNTPs, SYBR Green I dye), 0.3 μM of forward and reverse primers, 2-μL cDNA, and nuclease-free water. Previously established primers were used (Alteri and Mobley, 2007; Han et al., 2013). Real-time PCR was performed under the following cycle conditions: denaturation/polymerase activation at 95°C for 10 min and 45 cycles of denaturation at 95°C for 15 s, annealing at 59°C (*fyuA*) or 55°C (*iutA*, *iroN*, and *ireA*) or 58°C glyceraldehyde-3-phosphate dehydrogenase (*GAPDH*) for 30 s, extension at 72°C for 30 s. Specificity for all amplicons was confirmed via melting curves and electrophoresis in 2% agarose gels. No-template control (NTC) and RT controls were included in each assay. The reference gene encoding *GAPDH* was used to normalize the expression level of the analyzed genes. Relative expression values were measured using a standard curve analysis for each gene. The normalization values for the individual samples were compared to the control uropathogenic *Escherichia coli* (UPEC) strains CFT073 and CIP 105986 and expressed as the fold change. For the *ireA* gene, a commensal strain, showing expression levels for the remaining genes (*fyuA*, *iutA*, and *iroN*) similar to the control UPEC strains, was used as a control for normalization. Each cDNA synthesis and real-time PCR was performed in triplicate.

### Multilocus sequence typing

The *E. coli* isolates that contained at least one PAI were characterized by MLST. Analysis was performed using the seven-locus Achtman scheme. The internal fragments of seven housekeeping genes (*adk*, *fumC*, *gyrB*, *icd*, *mdh*, *purA*, and *recA*) were amplified by PCR with primers previously described (Wirth et al., 2006). The reaction conditions were as follows: 3 min at 94°C, 30 cycles of 1 min at 94°C, 1 min at annealing temp, 1 min at 72°C, followed by 5 min at 72°C. The PCR reaction mixture in a volume of 50 μL contained: buffer solution (Thermo Scientific, Waltham, MA, USA), 2.5-mM MgCl^2^ (Promega, Madison, WI, USA), 0.5 mM of each dNTP (Promega), 0.2 μM of each primer (Genomed, Poland), 1 U of Dream Taq Green DNA Polymerase (Thermo Scientific), and 6 μL of DNA template. The amplicons were purified and analyzed by Sanger sequencing (Genomed, Warszawa, Poland). The amplification primer pairs were used for the sequencing step. The resultant sequences were imported into the *E. coli* MLST database website (https://pubmlst.org/bigsdb?db=pubmlst_escherichia_seqdef&page=sequenceQuery) to determine sequence types (STs) and clonal complexes (ST Cplx) (Jolley et al., 2018) and confirmed on the website (https://enterobase.warwick.ac.uk/species/ecoli/search_strains?query=st_search).

### Statistical analysis

The classification to phylogroups (in categories A, B1, B2, C, D, E, F, clade I, and NT) presence of the PAIs, ability to biofilm formation (in categories S, M, and W), and hemolystic activity were categorized as 1 = present and 0 = absent. To determine whether there was a significant association between the phylogroups distribution, prevalence of the PAI, ability to form biofilm, hemolytic activity, and the host (adults vs. young children) Pearson’s chi-squared test for independence or Fisher’s exact test was used. The Fisher’s exact test was used if more than 20% of the cells in the contingency table have expected frequencies below five.

The evaluations of the frequency of the PAIs combinations, siderophore receptor genes combinations, and gene combinations within type 1 and P fimbriae operons among the *E. coli* isolates from adults and young children were tested using the chi-squared test for proportions or Fisher’s exact test for proportions. Fisher’s exact test was used when the assumptions of the chi-squared test did not hold. The null hypothesis assumes that the proportions in isolates from adults and young children are equal. The alternative hypothesis is one-sided and assumes that the proportion in one group of humans (adults or young children) was lower or higher than in the other, as appropriate. To control the number of false positive results in a series of tests for comparing two proportions, the method of false discovery rate (FDR) was used. The FDR Benjamini-Hochberg procedure (1995) allowed us to adjust the *p*-value in multiple testing (Benjamini and Hochberg, 1995).

For all the statistical tests, the level of statistical significance was defined as 0.05. The statistical analyses were performed using the program R (R Core Team, Vienna, Austria) (The R Core Team, 2024).

The distributions of relative expression of siderophore receptor genes *fyuA*, *iutA*, *iroN*, and *ireA* among *E. coli* isolates collected from adults in ST groups were presented using box plots.

A maximum-likelihood tree, depicting the phylogenetic relationships among the *E. coli* isolates from adults and young children, based on the nucleotide sequences of seven housekeeping genes: *adk*, *fumC*, *gyrB*, *icd*, *mdh*, *purA*, and *recA*. The tree was reconstructed with Randomized Axelerated Maximum Likelihood (RAxML), using a general time-reversible nucleotide substitution model GTRGAMMA and 1,000 bootstrap replicates (Chevenet et al., 2006; Kozlov et al., 2019; Guangchuang, 2022).

## Results

### Extended phylogenetic assignment of *E. coli*

An extended phylogenetic group classification indicated a significant difference in the phylogenetic structure of *E. coli* isolates from adults compared to those from young children (*p* < 0.001) (Supplementary Figure S2). Phylogroup B2 isolates were dominant among *E. coli* from adults (49.7%), followed by group A (17.5%) and D (13.3%); the phylogroups B1, C, E, and F occurred with a lower frequency. 1.4% of the isolates did not belong to any known phylogroup and, therefore, were assigned as not typeable (NT). Phylogroup A (68%) was the most frequent in the isolates from young children, followed by B1 (12%), while phylogroups B2, F, and clade I were identified at a lower frequency. 2% of isolates were classified as NT. Phylogenetic groups B2 and D were significantly more common among the isolates from adults than young children, *p* < 0.0001 and *p* = 0.008, respectively. Phylogenetic group A was significantly more frequent in isolates from young children than adults (*p* < 0.0001).

### Distribution of PAIs

**Figure 1:**
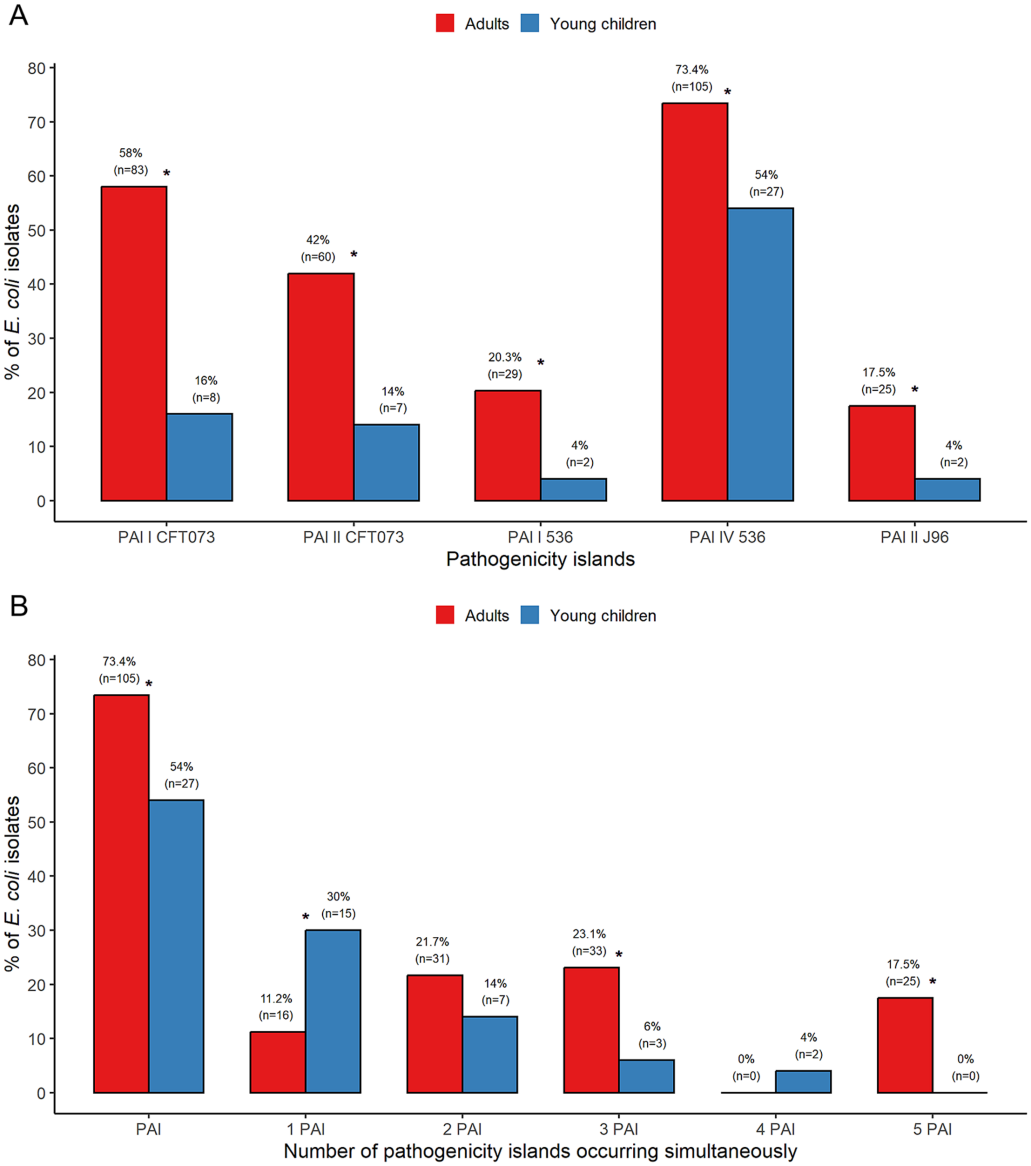
Analysis of the prevalence of PAIs **(A)** and the number of PAIs **(B)** among commensal *Escherichia coli* from adults and young children. *Statistically significant.

All five tested PAIs, PAI I CFT073, PAI II CFT073, PAI I 536, PAI IV 536, and PAI II J96, were identified in *E. coli* isolates from adults and young children. Each of the PAIs was significantly more common in *E. coli* from adults than from children, with *p* < 0.001 for PAI I CFT073, PAI II CFT073, and *p* < 0.05 for PAI I 536, PAI IV 536, and PAI II J96 (Figure 1A). Overall, significantly more *E. coli* isolates from adults (73.4%) than from young children (54%) harbored PAIs (*p* < 0.05). All these isolates carried PAI IV 536, the most frequently detected PAI in this study. Single PAI was present more regularly among isolates from young children (30%) than from adults (11.2%) (*p* < 0.01). Conversely, multiple PAIs were significantly more prevalent in *E. coli* from adults (62,2%) than from young children (24%) (*p* < 0.0001). Among isolates positive for multiple PAIs, the most frequent were *E. coli* carrying three PAIs simultaneously from adults (23.1%) (Figure 1B).

Among *E. coli* from adults, all isolates (100%) were classified into phylogroups B2 and F, which accumulated PAIs. The majority of phylogroup D isolates (73.3%) carried PAIs, whereas among the isolates of phylogroups A, B1, and E, the presence of PAIs was less frequent (44, 25, and 10%, respectively). The PAIs were not identified in phylogroup C and NT isolates. PAI I CFT073 and PAI IV 536 occurred in all *E. coli* isolates of phylogroups B2 and F from adults (Table 1).

**Table 1:** Distribution of PAIs among commensal *Escherichia coli* isolates from adults and young children, classified according to phylogenetic group.

Phylogenetic group	Number (%) of isolates with PAIs					
	PAIs	PAI I CFT073	PAI II CFT073	PAI I 536	PAI IV 536	PAI II J96
Adults						
A	11 (44)	1 (4,0)	2 (8)	-	11 (44)	-
B1	2 (25)	-	-	-	2 (25)	-
B2	71 (100)	71 (100)	52 (73.2)	28 (39.4)	71 (100)	24 (33.8)
C	-	-	-	-	-	-
D	14 (73.3)	4 (21.1)	5 (26.3)	-	14 (73.7)	-
E	1 (10)	1 (10)	1 (10)	1 (10)	1 (10)	1 (10)
F	6 (100)	6 (100)	-	-	6 (100)	-
NT	-	-	-	-	-	-
Young children						
A	11 (32.4)	1 (2.9)	3 (8.8)	-	11 (32.4)	-
B1	6 (100)	-	-	-	6 (100)	-
B2	4 (100)	4 (100)	2 (50)	2 (50)	4 (100)	2 (50)
F	3 (100)	1 (33.3)	2 (66.7)	-	3 (100)	-
Clade I	2 (100)	2 (100)	-	-	2 (100)	-
NT	1 (100)	-	-	-	1 (100)	-

In *E. coli* from young children, all isolates (100%) assigned to phylogroups B1, B2, F, and clade I harbored PAIs. The only isolate classified as NT contained PAI IV 536. However, PAIs were less frequent in phylogroup A (32.4%). PAI I CFT073 and PAI IV 536 were found in all *E. coli* phylogroups B2 and clade I isolates from young children (Table 1). All five tested PAIs occurred in phylogroups B2 and E (Table 1).

### Genotypic and phenotypic examination of the type 1 and P fimbria operons

The complete set of six tested genes (*fimB*, *fimE*, *fimA*, *fimI*, *fimC*, and *fimH*) of the type 1 fimbrial operon was detected in 54.5 and 40% of *E. coli* isolates from adults and young children, respectively. The isolates with complete operon and switch element in the “ON” orientation occurred with similar frequencies, 39.2 and 38%, among *E. coli* from adults and young children, respectively. The incomplete operon of the type 1 fimbria was identified in 43.4 and 52% of *E. coli* from adults and young children, respectively. The isolates without one gene were the most common in both groups, with 33.6 and 38% rates. The absence of the *fimA* gene was most frequently identified. *E. coli* isolates without two, four, and five genes within the type 1 fimbrial operon occurred less frequently (Supplementary Figure S3).

91.1 and 89.5% of isolates from adults and young children, with complete operon and switch element in the “ON” orientation, revealed mannose-sensitive yeast agglutination (MSYA).

Analysis of the P fimbria operon revealed a complete set of four tested genes (*papAH*, *papC*, *papEF*, and *papG*) in 13.3% of *E. coli* from adults and 12% from young children. The P fimbria operon was incomplete in 5.6% of *E. coli* isolates from adults; the absence of one or two genes was observed (Supplementary Figure S4). Among isolates with a complete operon from adults, the *papGIII* variant was dominant (78.9%), whereas in children, both the *papGI* and *papGIII* variants occurred with a frequency of 50% (Table 2).

**Table 2:** Prevalence of P fimbriae *papG* variants among commensal *Escherichia coli* isolates from adults and young children.

Number (%) of *Escherichia coli* with complete P fimbriae operon		
P fimbriae papG variant	Adults *n* = 19	Young children *n* = 6
*papGI*	0	3 (50)
*papGII*	4 (21.1)	0
*papGIII*	15 (78.9)	3 (50)

Expression of P fimbriae was monitored by agglutination of sheep erythrocytes among isolates with a complete P fimbriae operon. Mannose-resistant hemagglutination (MRHA) was detected in 78.9 and 50% of *E. coli* from adults and young children, respectively. All the *E. coli* isolates carrying the *papGIII* variant were positive in the MRHA test, whereas the isolates with *papGI* and *papGII* variants were negative.

### Biofilm formation and hemolytic activity

The majority of the *E. coli* isolates, 54.6% from adults and 68% from young children, showed moderate to strong biofilm formation ability. Strong biofilm producers were detected at similar frequencies, 19.6 and 22%, among *E. coli* isolates from adults and young children, respectively (Supplementary Figures S5A,B).

The *hlyA* gene was detected in 30 (21%) of *E. coli* isolates from adults and 3 (6%) from young children. Significantly more *E. coli* from adults 29 (20.3%) than from young children 2 (4%) showed hemolytic activity on blood agar (*p* < 0.01) (Supplementary Table S1).

### Evaluation of the simultaneous presence of several siderophore receptor genes

Genotypic screening of four siderophore receptor genes: yersiniabactin (*fyuA*), aerobactin (*iutA*), salmochelin (*iroN*) and TonB-dependent receptor (*ireA*) revealed that *E. coli* isolates with concurrent presence of two and three receptor genes was the most common among adults with frequency of 38.5 and 24.5%, respectively, compared to 8 and 4% among isolates from young children (*p* < 0.0005, *p* < 0.005). The isolates carrying one receptor gene were the most frequent (32%) among *E. coli* from young children, compared to adults (12.6%) (*p* < 0.005) (Supplementary Figure S6).

### Siderophore receptors expression

**Figure 2:**
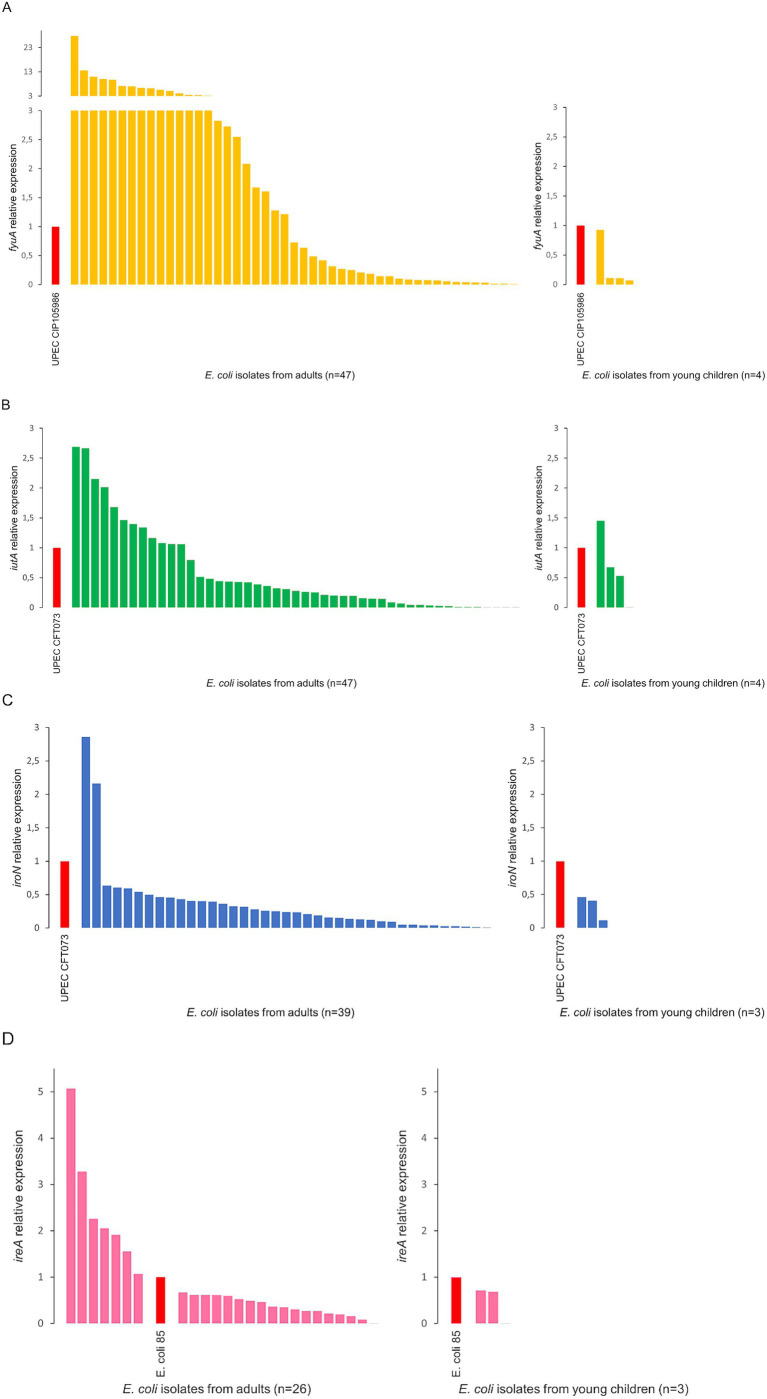
Analysis of the relative expression level of the siderophore receptor genes *fyuA* **(A)**, *iutA* **(B)**, *iroN* **(C)**, *ireA* **(D)** among *Escherichia coli* isolates from adults and young children. *E. coli* 85—an isolate of commensal *E. coli* from our collection, showing expression levels for the remaining genes (*fyuA*, *iutA*, and *iroN*) similar to the control UPEC strains—was used as a control for normalization.

Isolates with three siderophore receptor genes (35 and 2) or four (15 and 2) from adults and young children, respectively) were selected for this study. *E. coli* isolates revealed simultaneous expression of several siderophore receptor genes. The expression was characterized by varying levels, depending on the gene analyzed and the reference to the control UPEC strain. Three *E. coli* isolates from adults showed simultaneous expression of three receptor genes at levels higher than in control UPEC strains. Analysis revealed a higher expression level of the *fyuA* gene in 48,9% of *E. coli* from adults compared to the control UPEC CIP 105986 strain (Figure 2A). In the case of *iutA* and *iroN* genes, the expression was higher in 25.5 and 5.1% of isolates from adults, respectively, compared to the control UPEC CFT073 strain (Figures 2B,C). The expression of the *ireA* gene was higher compared to the commensal control strain in 26.9% of *E. coli* from adults (Figure 2D). Among the isolates from young children, lower expression of the *fyuA*, *iroN*, and *ireA* genes was observed compared to the control UPEC strains (Figures 2A,C,D). 25% of *E. coli* from young children showed a higher expression level of the *iutA* gene than the control UPEC CFT073 strain (Figure 2B).

### MLST analysis

**Figure 3:**
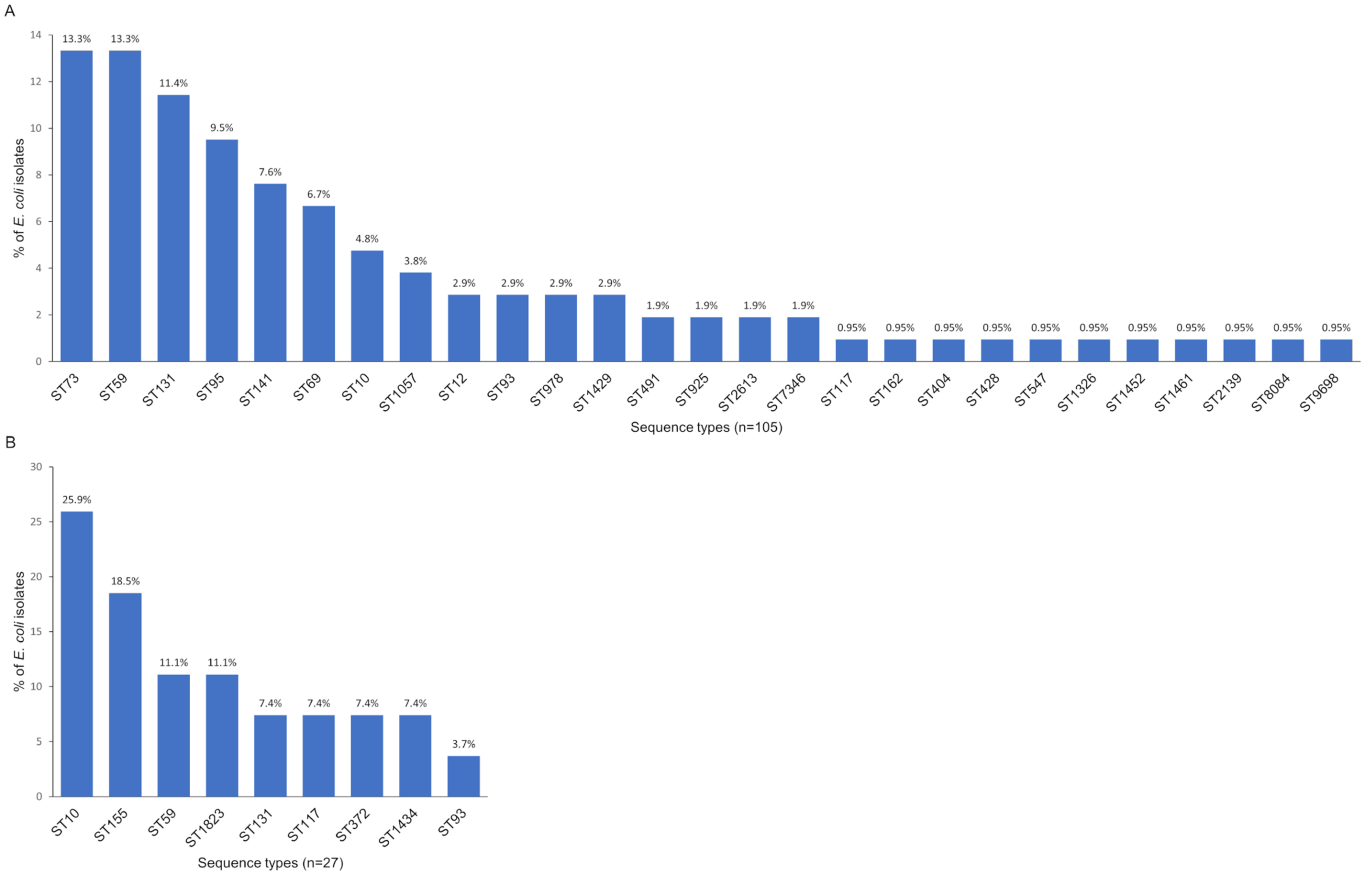
Distribution of the STs among commensal *Escherichia coli* isolates from adults **(A)** and young children **(B)**.

Our MLST analysis included *E. coli* isolates containing at least one PAI. We examined 105 isolates from adults, which were distributed among 27 STs. The most frequent STs were ST73, ST59 (both 13.3%), ST131 (11.4%), ST95 (9.5%), ST141 (7.6%), and ST69 (6.7%). The remaining STs occurred with a frequency below 5% (Figure 3A). We also analyzed 27 isolates from young children, which were assigned to 9 STs. The most common were ST10 (25.9%), ST155 (18.5%), ST59, and ST1823 (both 11.1%) (Figure 3B).

### Associations between ST and the level of siderophore receptor expression

**Figure 4:**
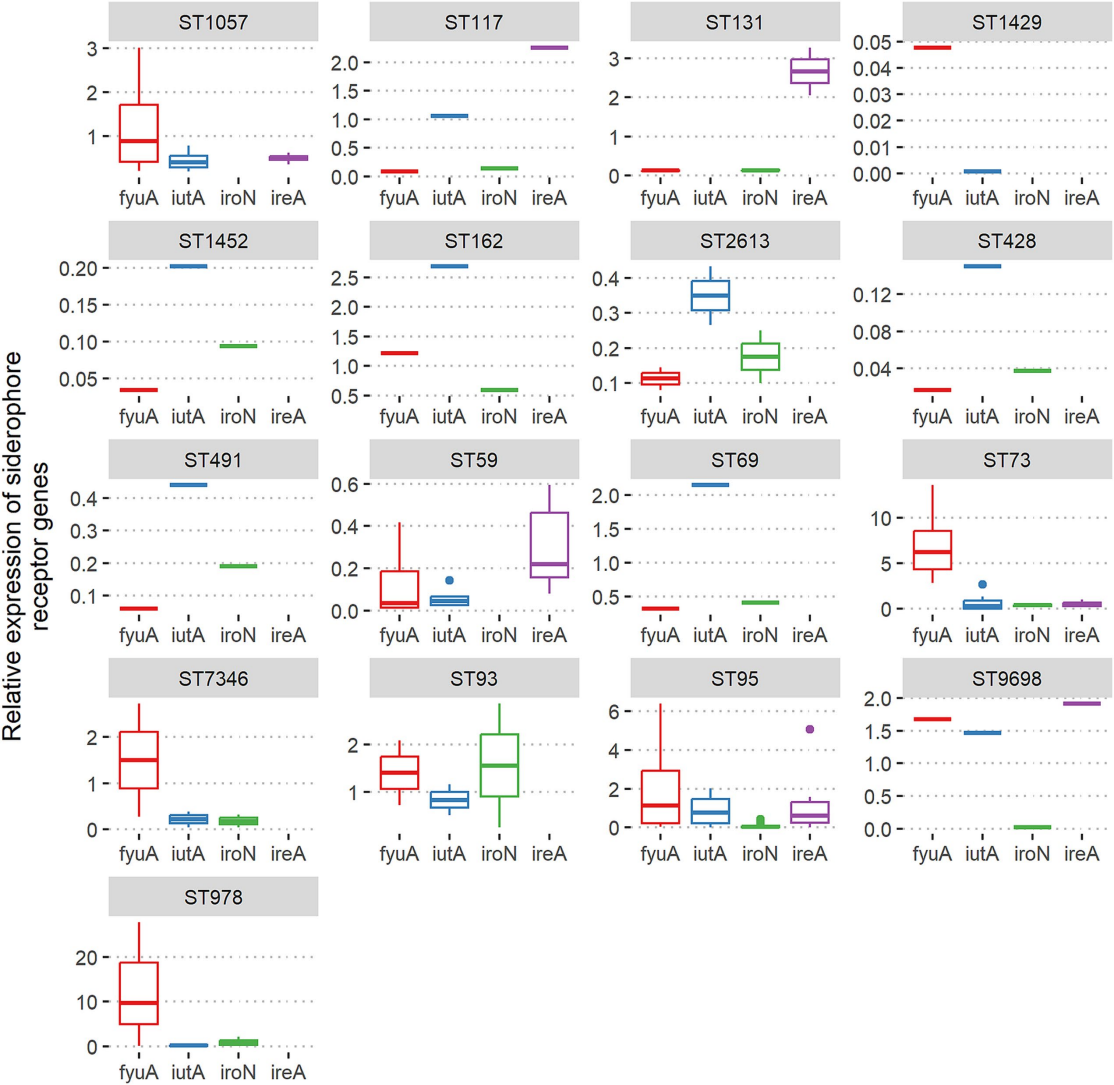
Associations between ST and relative expression of siderophore receptor genes *fyuA*, *iutA*, *iroN*, and *ireA* among *Escherichia coli* isolates from adults.

The highest level of *fyuA* gene expression was detected for ST978, ST73, ST95, ST7346, ST93, ST9698, and ST162. The *iutA* receptor high expression correlated with ST162, ST69, ST73, ST93, ST95, ST9698, and ST117. Significant expression level of the *iroN* receptor gene revealed only ST93 isolates. The highest expression of the *ireA* receptor gene was associated with ST131, ST117, ST73, ST95, and ST9698 (Figure 4).

### Relationships between sequence type lineages (ST), phylogeny, and genotypic and phenotypic traits of virulence

**Figure 5:**
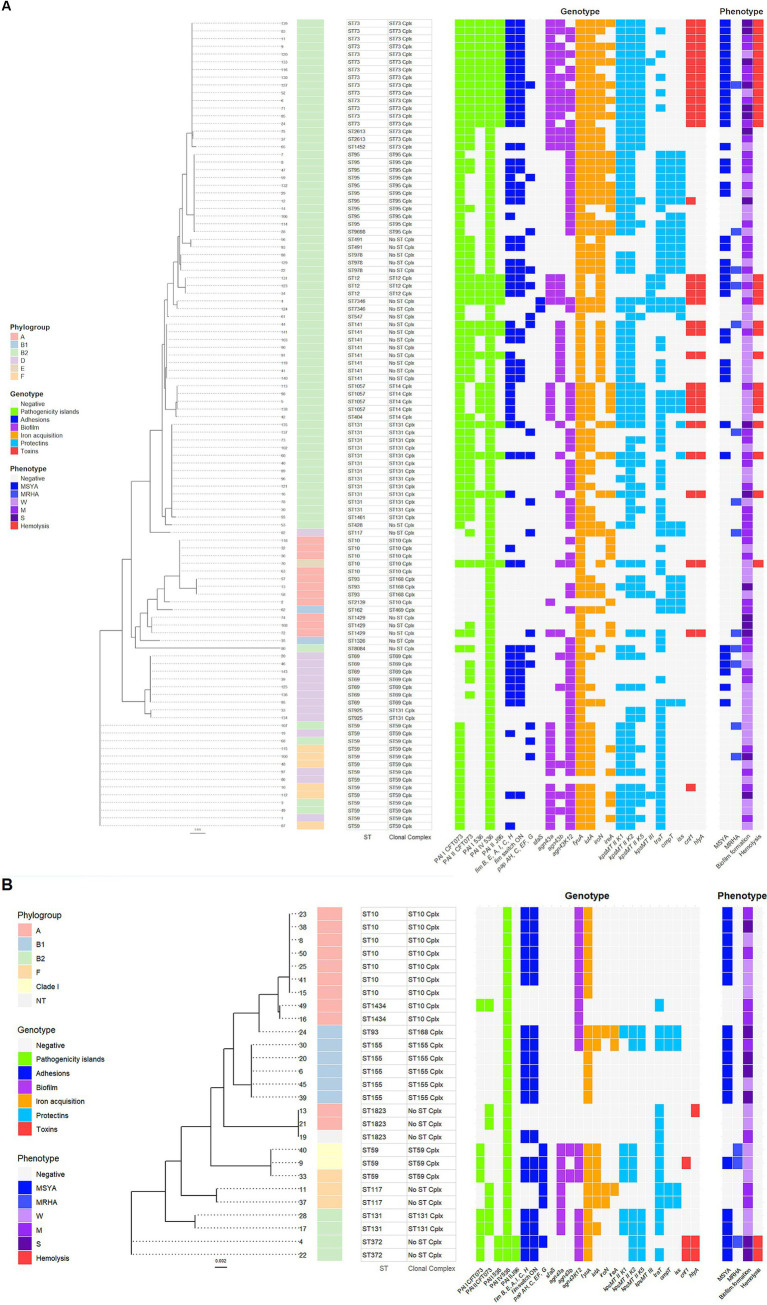
A maximum-likelihood tree depicting the phylogenetic relationships based on the nucleotide sequences of seven housekeeping genes: *adk*, *fumC*, *gyrB*, *icd*, *mdh*, *purA*, and *recA* among the *Escherichia coli* isolates from adults **(A)** and young children **(B)**. The tree was reconstructed with RAxML, using a general time-reversible nucleotide substitution model (GTRGAMMA) and 1,000 bootstrap replicates. The isolates’ names are indicated at the tip of the tree. Next, the phylogroups with the color displayed in the legend, STs, and clonal complexes are presented. A parts heat map is given on the right side of the figure. The first part, designated as genotype, encompasses PAIs and the VGs clustered according to their function and marked with different colors displayed in the legend. The second part, designated as phenotype, presents phenotypic traits of virulence, marked with colors corresponding to the genotype in the legend. The degree of biofilm formation was classified into three categories: S, strong; M, moderate; and W, weak.

*Escherichia coli* isolates from adults carrying PAIs were mainly classified as phylogroup B2. The maximum likelihood tree based on the nucleotide sequences of seven housekeeping genes saw the isolates of phylogroup B2 cluster in several STs, with the most numerous being ST73, ST95, ST978, ST12, ST141, ST1057, and ST131 (Figure 5A). All ST73 and ST12 isolates carried five PAIs, a complete type 1 fimbria operon with switch ON and the ability to MSYA, and the toxins *cnf1* and *hlyA* genes with the ability to hemolyze. ST73 isolates stand out among other STs regarding the presence and phenotypic expression of the most extraintestinal virulence factors. ST73 *E. coli* also harbored *agn43a* or *a* and *b* variants (most of them with strong or moderate biofilm formation ability), *kpsMTII* variants *K1*, *K2*, and *K5*, three or four siderophore receptor genes (78.6%), some of them with a significant level of expression. The part of ST141 (37.5%) and ST131 (25%) isolates carried five PAIs, toxins *cnf1* and *hlyA*, with the ability to hemolyze. Two ST131 isolates (16.7%) revealed similar to ST73 *E. coli* extraintestinal virulence potential with the presence of genes involving adhesion, biofilm formation (*agn43b*), iron acquisition, and capsular antigens-related genes, as well as the ability to MSYA, strong/moderate biofilm formation, and high *ireA* expression. The isolate ST7346 is also interesting in terms of its extraintestinal virulence potential (5 PAIs, *sfaS* fimbriae gene, all protectins and toxins genes) with moderate ability to form biofilm, high level of *fyuA* expression, and hemolytic activity. The last isolate with five PAIs was classified as phylogroup E, represents ST10, and revealed hemolytic activity without MSYA ability. The remaining PAIs harboring *E. coli* isolates from adults were grouped into several ST clusters. Phylogroup A was represented mainly by ST10, ST93, and ST1429, the majority of them carrying one PAI. Phylogroup D gathered *E. coli* isolates of ST69 and ST59 with one or two PAIs. Isolates assigned to phylogroup F were represented by ST59 and loaded by two PAIs (Figure 5A).

PAIs harboring *E. coli* isolates from young children cluster in nine STs, with the dominance of phylogroup A (Figure 5B). Four isolates of ST372 and ST131 represented phylogroup B2. *E. coli* ST372 revealed the highest extraintestinal virulence potential, carrying four PAIs, a complete type 1 fimbria operon with switch ON, toxins *cnf1* and *hlyA* genes, and showed the ability to MSYA, strong biofilm formation, and hemolysis. ST131 *E. coli* contained three PAIs and showed the ability to MSYA but lacked toxin genes. Isolates classified to phylogroup F belonged to ST117 and ST59 and carried two PAIs. Phylogroup A was mainly represented by ST10 isolates with one PAI and showed MSYA ability. Phylogroup B1 mainly gathered isolates of ST155, carrying one PAI, and showed MSYA ability. Two isolates of ST59 with two PAIs and the ability to MRHA were assigned to clade I (Figure 5B).

## Discussion

This study represents an essential contribution to understanding the genetic structure and the relationship between genetic background and phenotypic profile of selected virulence factors in two collections of commensal *E. coli* isolates from healthy adults and young children. The analyses revealed the associations between the ST and extraintestinal virulence traits. Commensal *E. coli* constitute the primary reservoir of opportunistic extraintestinal pathogens, and our study enabled us to monitor the share of strains with ExPEC features in the population. The phylogenetic structure analysis revealed complex phylogeny in both groups of isolates from adults and young children. The main phylogenetic pattern of this classification, with significant dominance of phylogroup B2 in isolates from adults and phylogroup A in young children, is consistent with our previous study of similar populations of *E. coli* isolates (Bok et al., 2018). The presence of phylogroups C, D, and E only among isolates from adults and clade I in young children further emphasized the complexity. It was reported that between 1980 and 2010, the proportion of phylogroup B2 isolates in many industrialized countries increased significantly (Tenaillon et al., 2010; Marin et al., 2022). We observed a similar tendency in the increase of B2 phylogroup rates, comparing our previous research (Bok et al., 2018), where 46.6% of *E. coli* were assigned to phylogroup B2 (2015), to 49.7% in this study. The predominance of phylogroup A among young children probably results from differences in the dietary habits between adults and children. The diet of young children aged 0.5–3 years is relatively simple and primarily consists of organic, less processed foods than the adult diet.

PAIs are considered essential elements in the bacterial genome, playing a significant role in the pathogenicity and evolution of ExPEC (Brzuszkiewicz et al., 2006; Desvaux et al., 2020), though they have received little attention in commensal strains (Sabaté et al., 2006; Li et al., 2010; Östblom et al., 2011). This study showed that all five tested PAIs: PAI I CFT073, PAI II CFT073, PAI I 536, PAI IV 536, and PAI II J96 occurred among *E. coli* isolates from adults and young children. Significantly more *E. coli* isolates from adults (73.4%) than young children (54%) carried PAIs. Our investigation also revealed that all *E. coli* representing phylogroups B2 and F harbored PAIs in adults and young children. In contrast, PAIs were less frequent in phylogroup A (44 and 32.4%, respectively). Earlier studies indicated lower rates, 40% (Sabaté et al., 2006) and 46.8% (Li et al., 2010) of PAI occurrence in commensal *E. coli*, which agrees with the proportion of PAIs in our isolates from young children. This resulted from the lower share of phylogroup B2 in these *E. coli* populations compared to the adult population in this study. Moreover, it was also reported that the prevalence of virulence genes increased between 1980 and 2010, driven by the rise in frequency of phylogroup B2, carrying numerous virulence factors (Marin et al., 2022). The most prevalent PAI in our study was PAI IV 536, similar to other works’ results (Sabaté et al., 2006; Li et al., 2010; Östblom et al., 2011). This PAI was also frequently found alone in isolates from non-B2 groups and has been the most widespread PAI in *Enterobacteriaceae*, more linked to fitness than pathogenicity (Schubert et al., 2004). It has been documented that the accumulation of PAIs, with their virulence factors, in individual isolates is positively associated with their duration of persistence in the colon (Nowrouzian et al., 2001b, 2001a; Östblom et al., 2011).

Among the genes collected on PAIs, fimbria genes are one of the most pervasive. Type 1 and P fimbriae are the primary virulence factors of UPEC strains. Type 1 fimbriae are responsible mainly for bladder infection; in turn, P fimbriae are related to pyelonephritis (Desvaux et al., 2020). Our study indicated that 97.9 and 92% *E. coli* isolates from adults and young children carried genes from the type 1 fimbria operon region. Still, deletion of one or more genes was found in many isolates. Complete operon was identified in 54.5 and 40% of isolates from adults and young children, respectively, with the promoter in the “ON” position (active promoter) in 39.2 and 38%. Our earlier results indicated a similar proportion (56.7%) of commensal *E. coli* with complete type 1 fimbria operon and 41.7% with switch element in the “ON” orientation (Pusz et al., 2014). Phenotypic analysis of type 1 fimbriae expression showed that not all, but the vast majority (91.1 and 89.5% from adults and children, respectively) of these isolates were positive in the MSYA test. These results indicated that the presence of the *fimH* gene (often used as a marker of type 1 fimbriae) does not reflect the expression status of type 1 fimbriae, because in the case of some isolates, part of the type 1 fimbria operon is often deleted, which prevents the expression of this trait. The complete P fimbriae operon proportion was much lower than in type 1 fimbriae (13.3 and 12% of *E. coli* from adults and young children, respectively). Notably, 78.9 and 50% of these isolates were positive in the MRHA test, which shows that the presence of a gene/genes in an operon may not always indicate that a given feature will be subject to phenotypic expression. Only the *E. coli* isolates carrying the *papGIII* variant were positive in the MRHA test. The *GIII* allele is found mainly in strains isolated from cystitis (Desvaux et al., 2020). The ability to adhesions (MSYA and MRHA) was not correlated with specific phylogroups. The presence of fimbriae can be considered an adaptation of commensal strains within the intestinal microbiome community (Nowrouzian et al., 2001b, 2001a; Dobrindt et al., 2013). Interestingly, it was reported that expression of type 1 fimbrial genes was among the most highly expressed genes during murine experimental UTI. Still, only 25% of *E. coli* isolates expressed type 1 fimbrial genes in urine collected from cystitis patients. Despite the lack of type 1 fimbrial expression in the urine samples, these *E. coli* isolates were generally capable of expressing type 1 fimbriae *in vitro*, possibly because adhering bacteria are not released into the urine. Adhesin genes encoding P fimbriae were expressed at very low levels during *in vitro* culture and infection (Hagan et al., 2010; Sintsova et al., 2019).

In our study, we observed similar frequencies of *E. coli* from adults and young children in three categories of biofilm formation: weak, moderate, and strong. The strong biofilm formation ability was observed with 19.6 and 22% rates among isolates from adults and young children, respectively, and this feature is not correlated with a specific phylogenetic group. The biofilm formation depends on many factors, like type 1 fimbriae and P fimbriae, which play a crucial role in the initial adhesion to surfaces, as well as flagella, Ag43, and exopolymeric substances. Moreover, environmental conditions such as nutrient availability can enhance biofilm formation by providing the necessary resources for bacterial growth. There was no association of increased biofilm formation *in vitro* with a strain collection representing pathogenic *E. coli* strains. The genetic and environmental factors influencing the biofilm phenotype in *E. coli* are very complex (Reisner et al., 2006). Unlike biofilm formation, hemolytic activity correlated strongly with the B2 phylogroup, with the difference in hemolysis ability between adults (20.3%) and children (4%) arising from the distribution of phylogenetic groups. The majority of the *E. coli* positive for the *hlyA* gene (29/30 and 3/4 from adults and young children, respectively) showed hemolytic activity on blood agar. Producing *α*-hemolysin requires the coordinated expression of several genes within the *hlyCABD* operon. Thus, the lack of hemolytic activity may be related to deletions or point mutations in one of these genes (Sobieszczańska, 2007; Burgos and Beutin, 2010).

This study showed that the presence of siderophore receptor genes is a common feature of isolates from adults (86%). In contrast, in *E. coli* from children, it occurs with about twice the lower frequency (48%). It was also revealed that several receptor genes often occurred simultaneously among isolates from adults (38.5 and 24.5% with two and three genes, respectively). Our earlier study indicated that the occurrence of genes for siderophore receptors was strongly associated with the B2 phylogroup in both adult and young children isolates and also with B1 and D in children (Bok et al., 2018). In this study, the differences in the frequencies of siderophore receptor genes in isolates from adults and young children result from the much lower share of phylogroup B2 in children. The *fyuA* gene was observed to be the most prevalent siderophore receptor gene among *E. coli* from adults and young children. The frequencies of occurrence of the *fyuA* approximately corresponded to the proportions of the PAI IV 536, which harbors the yersiniabactin iron acquisition system.

Our research indicates that when multiple siderophore receptor genes are present simultaneously, none are preferentially selected; all of them can be expressed in individual isolates, even though the metabolic cost of generating siderophores is significant and has a noticeable impact on the *E. coli* metabolome. Depending on the specific isolate and analyzed gene, expression occurred at different levels. The *fyuA* gene represents the broadest range of expression levels. Moreover, most commensal isolates exhibited higher expression of the *fyuA* gene (48.9%) than the UPEC control strain. In turn, *iutA* and *iroN* genes were less frequently expressed (25.5 and 5.1%, respectively) above the UPEC control. Other studies comparing siderophore production among fecal and plant-associated strains showed a wide range of siderophore production levels, with significant differences in enterobactin production, observable between plant-associated and fecal isolates at the population level (Searle et al., 2015). In contrast to our results in the mentioned study, low yersiniabactin gene expression level was detected. This indicated considerable differences in siderophore expression regulation among individual *E. coli* isolates, influenced by genetic background and environmental factors such as nutrient availability. The other reports also showed that rectal *E. coli* isolates can simultaneously produce more than one siderophore and have been observed to produce yersiniabactin, salmochelin, and aerobactin. Two siderophores, yersiniabactin and salmochelin, were produced more significantly among UTI strains compared to rectal, whereas aerobactin production was not preferentially associated with urinary strains (Henderson et al., 2009). We are aware that *in vitro* assays never fully reflect *in vivo* conditions. The host environment, including immune responses, nutrient availability, and physical interactions, often influences gene expression in ways that cannot be entirely replicated *in vitro*. Nevertheless, *in vitro* studies are usually the first step before more advanced research and can provide valuable information on the expression of virulence factors. As previously reported, the genes involved in iron acquisition in UPEC were highly expressed during both *in vitro* urine culture and *in vivo* during murine and human UTI. There was also a very good correlation comparing relative expression of iron acquisition genes during human versus murine UTI, which are highly expressed (Hagan et al., 2010; Subashchandrabose et al., 2014; Mobley, 2016; Sintsova et al., 2019). Although we cannot directly compare our results to those obtained from urine samples of patients with UTI (Hagan et al., 2010; Mobley, 2016), a specific trend regarding gene expression of individual siderophores is evident from the available data and the results presented here, namely the *fyuA* gene is most frequently expressed at the highest levels, followed by the *iutA* gene, while high expression of the *iroN* gene is rarely observed.

Many studies on ExPEC lineages focus on isolates from UTI or bloodstream infections, while only a few involve *E. coli* isolates from colonization. Therefore, our results will help supplement the information regarding the global distribution of commensal *E. coli* ST lineages and, for the first time, provide data from Poland (Central Europe region). A recent systematic review indicated that a subset of 20 pathogenic *E. coli* lineages: ST131, ST69, ST10, ST405, ST38, ST95, ST648, ST73, ST410, ST393, ST354, ST12, ST127, ST167, ST58, ST617, ST88, ST23, ST117, and ST1193 are responsible for the majority of ExPEC infections. MLST is the most widely used method for identifying them (Manges et al., 2019). Referring to the mentioned 20 STs closely related to ExPEC, among our isolates from adults, seven of them were identified: ST131, ST69, ST10, ST95, ST73, ST12, and ST117, which constituted 49.6% of the analyzed isolates. 27.6% of *E. coli* from adults belonged to STs, less common among ExPEC: ST59, ST141, ST93, ST491, ST162, and ST404. Strong ExPEC-associated ST10, ST131, and ST117 were identified in the collection of isolates from young children (40.7%). Types that are less common among ExPEC were: ST59, ST155, ST372, and ST93, which represented 40.7% of these *E. coli* isolates. We are aware of the limitations of these studies because only isolates carrying pathogenicity islands were analyzed. Nevertheless, these results provide important information regarding the commensal *E. coli* populations and allow for the analysis of the spreading of STs specific for ExPEC. The results of the present study point to ST73 as particularly distinguished from other STs in terms of extraintestinal virulence potential and being one of the most common in the adult commensal *E. coli* population. It carried the largest pool of virulence genes. Also, it exhibited phenotypic traits such as adhesion ability, moderate to strong biofilm formation ability, and hemolytic activity. Some ST73 isolates showed high-level expression of siderophore genes, particularly *fyuA*. The other STs with a slightly lower virulence potential but still significant were ST131, ST12, and ST141. An interesting case is ST7346 isolate, carrying five PAIs, *sfaS* fimbriae gene, all protectin and toxin genes, and revealed moderate ability to biofilm formation, high level of *fyuA* expression, and hemolytic activity, but so far not reported as ExPEC. As mentioned earlier, ST73, ST131, ST12, and ST141 are all strongly associated with UTI and bloodstream infections, but they also were identified in colonization studies (Manges et al., 2019; Bogema et al., 2020; Marin et al., 2022; Li et al., 2023). An extensive population analysis of 403 commensal strains from healthy adults in France showed the five most frequent STs were ST10, ST73, ST95, ST69, and ST59. ST141 and ST131 were also identified but less frequently (Marin et al., 2022). In our study, the most prevalent were ST73, ST59, ST131, ST95, ST141, and ST69 with frequencies above 5% among the *E. coli* from adults, and ST10, ST155, ST59, and ST1823 with proportions above 10% in isolates from young children. The greatest extraintestinal virulence potential among isolates from children was found for ST372, associated with adhesion ability (MSYA), strong biofilm formation, and hemolysis. It has been reported that this ST was isolated from newborns with early-onset sepsis and meningitis (Weissman et al., 2016). Among *E. coli* isolates from young children also identified ST131 as the second ST representing higher virulence potential than other STs.

It has been shown that the diversity of STs among commensal *E. coli* increased over time. This rise in ST diversity was attributed not to the increased frequency of B2 strains, but to the higher frequency of rare STs from 2001 to 2010. Moreover, higher virulence gene frequency evolved due to increased virulence gene frequency within STs and the clonal expansion of more virulent STs (Marin et al., 2022). VGs equip ExPEC for survival outside the gastrointestinal tract and contribute to its persistence in the human gut. Our study indicated that there is no difference between commensal isolates and ExPEC in terms of sequence type and the presence of virulence genes. Moreover, commensal *E. coli* showed phenotypic characteristics typical of ExPEC. These results confirm that the human gastrointestinal tract is the major reservoir of the ExPEC, and there may not be an absolute distinction between commensal *E. coli* and ExPEC. Moreover, they reveal interrelationships between ST, genetic background, and phenotypic expression of ExPEC virulence traits in commensal *E. coli*, and constitute the first such study for the Polish populations.

## Conclusion

Our results compared the virulence potential relevant for extraintestinal pathogenicity in two collections of commensal *E. coli* isolates from adults and young children. These two populations differ significantly regarding phylogeny and phenotypic expression of some extraintestinal virulence traits. *E. coli* from adults carried PAIs and VGs in a significantly higher proportion, resulting from the dominance of phylogroup B2 in this set of isolates. More common hemolytic activity and higher levels of the expression of siderophore receptors in *E. coli* from adults are closely related to the dominance of phylogroup B2. The other traits not associated with phylogroup B2, such as the ability to adhesion and strong biofilm formation tendency, were detected with similar frequency in both pools of isolates from adults and young children. The results indicated that during adolescence in humans, the commensal *E. coli* population inhabiting the digestive tract tends to change its phylogenetic structure toward the dominance of group B2 with its numerous virulence factors. STs associated with extraintestinal pathogenicity, such as ST73, ST131, ST10, and ST95, indicate these isolates as a potential source of endogenous infections. Our results highlight ST73 isolates from adults as possessing a distinct virulence potential. The similarities in phylogeny and virulence potential between commensals and ExPEC strains make it difficult to draw a clear boundary between these two groups. Further whole genome sequencing analysis and *in vivo* studies of the sepsis model of selected STs with the highest virulence potential would be advisable, especially isolates such as ST7346, which are not yet known as ExPEC and have shown high virulence potential in our studies. This study completes the map of the global distribution of ExPEC-typical STs in two commensal *E. coli* populations from adults and young children from Poland, which is essential for monitoring the geographic diversity of these strains. It is important to limit their spread by evaluating the transmission rate of these *E. coli* strains with ExPEC pathogenicity potential are also a part of the intestinal microbiome. Therefore, they cannot be eliminated; they can be only monitored.

## Data Availability

The original contributions presented in the study are included in the article/Supplementary material, further inquiries can be directed to the corresponding author.

## References

[ref1] Al Mayahie S. M. (2014). Phylogenetic grouping of dominant fecal *Escherichia coli* isolates from healthy males and females in Al-Kut/Wasit Province/Iraq. J. Bacteriol. Parasitol. 6:1–4. doi: 10.4172/2155-9597.1000215

[ref2] Alteri C. J. Mobley H. L. T. (2007). Quantitative profile of the Uropathogenic *Escherichia coli* outer membrane proteome during growth in human urine. Infect. Immun. 75, 2679–2688. doi: 10.1128/IAI.00076-06 17513849 PMC1932884

[ref3] Benjamini Y. Hochberg Y. (1995). Controlling the false discovery rate: a practical and powerful approach to multiple testing. J. Royal Statistical Society Series B 57, 289–300. doi: 10.1111/j.2517-6161.1995.tb02031.x

[ref4] Blumer C. Kleefeld A. Lehnen D. Heintz M. Dobrindt U. Nagy G.. (2005). Regulation of type 1 fimbriae synthesis and biofilm formation by the transcriptional regulator LrhA of *Escherichia coli*. Microbiology 151, 3287–3298. doi: 10.1099/mic.0.28098-0, PMID: 16207912

[ref5] Bogema D. R. McKinnon J. Liu M. Hitchick N. Miller N. Venturini C.. (2020). Whole-genome analysis of extraintestinal *Escherichia coli* sequence type 73 from a single hospital over a 2 year period identified different circulating clonal groups. Microbial Genomics 6:1–18. doi: 10.1099/mgen.0.000255, PMID: 30810518 PMC7067039

[ref6] Bok E. Mazurek J. Myc A. Stosik M. Wojciech M. Baldy-Chudzik K. (2018). Comparison of commensal *Escherichia coli* isolates from adults and young children in Lubuskie Province, Poland: virulence potential, phylogeny and antimicrobial resistance. IJERPH 15:617. doi: 10.3390/ijerph15040617, PMID: 29597292 PMC5923659

[ref7] Braz V. S. Melchior K. Moreira C. G. (2020). *Escherichia coli* as a multifaceted pathogenic and versatile bacterium. Front. Cell. Infect. Microbiol. 10:1–9. doi: 10.3389/fcimb.2020.548492, PMID: 33409157 PMC7779793

[ref8] Brzuszkiewicz E. Brüggemann H. Liesegang H. Emmerth M. Ölschläger T. Nagy G.. (2006). How to become a uropathogen: comparative genomic analysis of extraintestinal pathogenic *Escherichia coli* strains. Proc. Natl. Acad. Sci. USA 103, 12879–12884. doi: 10.1073/pnas.0603038103, PMID: 16912116 PMC1568941

[ref9] Burgos Y. Beutin L. (2010). Common origin of plasmid encoded alpha-hemolysin genes in *Escherichia coli*. BMC Microbiol. 10:193. doi: 10.1186/1471-2180-10-193, PMID: 20637130 PMC2918590

[ref10] Chapman T. A. Wu X.-Y. Barchia I. Bettelheim K. A. Driesen S. Trott D.. (2006). Comparison of virulence gene profiles of *Escherichia coli* strains isolated from healthy and diarrheic swine. Appl. Environ. Microbiol. 72, 4782–4795. doi: 10.1128/AEM.02885-05, PMID: 16820472 PMC1489375

[ref11] Chevenet F. Brun C. Bañuls A.-L. Jacq B. Christen R. (2006). TreeDyn: towards dynamic graphics and annotations for analyses of trees. BMC Bioinformatics 7:439. doi: 10.1186/1471-2105-7-439, PMID: 17032440 PMC1615880

[ref12] Clermont O. Christenson J. K. Denamur E. Gordon D. M. (2013). The C lermont *E scherichia coli* phylo-typing method revisited: improvement of specificity and detection of new phylo-groups. Environ. Microbiol. Rep. 5, 58–65. doi: 10.1111/1758-2229.12019, PMID: 23757131

[ref13] Clermont O. Gordon D. M. Brisse S. Walk S. T. Denamur E. (2011). Characterization of the cryptic *Escherichia* lineages: rapid identification and prevalence. Environ. Microbiol. 13, 2468–2477. doi: 10.1111/j.1462-2920.2011.02519.x, PMID: 21651689

[ref14] Dale A. P. Woodford N. (2015). Extra-intestinal pathogenic *Escherichia coli* (ExPEC): disease, carriage and clones. J. Infect. 71, 615–626. doi: 10.1016/j.jinf.2015.09.009, PMID: 26409905

[ref15] Denamur E. Clermont O. Bonacorsi S. Gordon D. (2021). The population genetics of pathogenic *Escherichia coli*. Nat. Rev. Microbiol. 19, 37–54. doi: 10.1038/s41579-020-0416-x 32826992

[ref16] Desvaux M. Dalmasso G. Beyrouthy R. Barnich N. Delmas J. Bonnet R. (2020). Pathogenicity factors of Genomic Islands in intestinal and Extraintestinal *Escherichia coli*. Front. Microbiol. 11:2065. doi: 10.3389/fmicb.2020.02065, PMID: 33101219 PMC7545054

[ref17] Dobrindt U. Agerer F. Michaelis K. Janka A. Buchrieser C. Samuelson M.. (2003). Analysis of genome plasticity in pathogenic and commensal *Escherichia coli* isolates by use of DNA arrays. J. Bacteriol. 185, 1831–1840. doi: 10.1128/JB.185.6.1831-1840.2003, PMID: 12618447 PMC150128

[ref18] Dobrindt U. Hacker J. H. Svanborg C. (2013). Between pathogenicity and commensalism. Heidelberg: Springer International Publishing. 10.1007/978-3-642-36560-7 23781553

[ref19] Frankel G. Ron E. Z. (2018). Escherichia coli, a Versatile Pathogen. Cham: Springer International Publishing.

[ref20] Gordon D. M. Stern S. E. Collignon P. J. (2005). Influence of the age and sex of human hosts on the distribution of *Escherichia coli* ECOR groups and virulence traits. Microbiology 151, 15–23. doi: 10.1099/mic.0.27425-0, PMID: 15632421

[ref21] Guangchuang Y. (2022). Data integration, manipulation and visualization of phylogenetic trees. 1st Edn. New York: Chapman and Hall/CRC.

[ref22] Hagan E. C. Lloyd A. L. Rasko D. A. Faerber G. J. Mobley H. L. T. (2010). *Escherichia coli* global gene expression in urine from women with urinary tract infection. PLoS Pathog. 6:e1001187. doi: 10.1371/journal.ppat.1001187, PMID: 21085611 PMC2978726

[ref23] Han X. Bai H. Liu L. Dong H. Liu R. Song J.. (2013). The luxS gene functions in the pathogenesis of avian pathogenic *Escherichia coli*. Microb. Pathog. 55, 21–27. doi: 10.1016/j.micpath.2012.09.008 23046700

[ref24] Henderson J. P. Crowley J. R. Pinkner J. S. Walker J. N. Tsukayama P. Stamm W. E.. (2009). Quantitative metabolomics reveals an epigenetic blueprint for Iron acquisition in Uropathogenic *Escherichia coli*. PLoS Pathog. 5:e1000305. doi: 10.1371/journal.ppat.1000305, PMID: 19229321 PMC2637984

[ref25] Hernandes R. T. Velsko I. Sampaio S. C. F. Elias W. P. Robins-Browne R. M. Gomes T. A. T.. (2011). Fimbrial Adhesins produced by atypical Enteropathogenic *Escherichia coli* strains. Appl. Environ. Microbiol. 77, 8391–8399. doi: 10.1128/AEM.05376-11, PMID: 21926222 PMC3233042

[ref26] Hou B. Meng X.-R. Zhang L.-Y. Tan C. Jin H. Zhou R.. (2014). TolC promotes ExPEC biofilm formation and Curli production in response to medium Osmolarity. Biomed. Res. Int. 2014, 1–10. doi: 10.1155/2014/574274, PMID: 25243151 PMC4163439

[ref27] Janben T. Schwarz C. Preikschat P. Voss M. Philipp H. Wieler L. H. (2001). Virulence-associated genes in avian pathogenic *Escherichia coli* (APEC) isolated from internal organs of poultry having died from colibacillosis. Int. J. Med. Microbiol. 291, 371–378. doi: 10.1078/1438-4221-00143, PMID: 11727821

[ref28] Johnson J. R. O’Bryan T. T. (2004). Detection of the *Escherichia coli* Group 2 polysaccharide capsule synthesis gene *kpsM* by a rapid and specific PCR-based assay. J. Clin. Microbiol. 42, 1773–1776. doi: 10.1128/JCM.42.4.1773-1776.2004, PMID: 15071046 PMC387594

[ref29] Johnson J. R. Stell A. L. (2000). Extended virulence genotypes of *Escherichia coli* strains from patients with Urosepsis in relation to phylogeny and host compromise. J. Infect. Dis. 181, 261–272. doi: 10.1086/315217, PMID: 10608775

[ref30] Jolley K. A. Bray J. E. Maiden M. C. J. (2018). Open-access bacterial population genomics: BIGSdb software, the PubMLST.org website and their applications. Wellcome Open Res 3:124. doi: 10.12688/wellcomeopenres.14826.1, PMID: 30345391 PMC6192448

[ref31] Kaper J. B. Nataro J. P. Mobley H. L. T. (2004). Pathogenic *Escherichia coli*. Nat. Rev. Microbiol. 2, 123–140. doi: 10.1038/nrmicro818, PMID: 15040260

[ref32] Koga V. L. Tomazetto G. Cyoia P. S. Neves M. S. Vidotto M. C. Nakazato G.. (2014). Molecular screening of virulence genes in Extraintestinal pathogenic *Escherichia coli* isolated from human blood culture in Brazil. Biomed. Res. Int. 2014, 1–9. doi: 10.1155/2014/465054, PMID: 24822211 PMC4009324

[ref33] Köhler C.-D. Dobrindt U. (2011). What defines extraintestinal pathogenic *Escherichia coli*? Int. J. Med. Microbiol. 301, 642–647. doi: 10.1016/j.ijmm.2011.09.006, PMID: 21982038

[ref34] Kozlov A. M. Darriba D. Flouri T. Morel B. Stamatakis A. (2019). RAxML-NG: a fast, scalable and user-friendly tool for maximum likelihood phylogenetic inference. Bioinformatics 35, 4453–4455. doi: 10.1093/bioinformatics/btz305, PMID: 31070718 PMC6821337

[ref35] Landraud L. Gibert M. Popoff M. R. Boquet P. Gauthier M. (2003). Expression of *cnf1* by *Escherichia coli* J96 involves a large upstream DNA region including the *hlyCABD* operon, and is regulated by the RfaH protein. Mol. Microbiol. 47, 1653–1667. doi: 10.1046/j.1365-2958.2003.03391.x, PMID: 12622819

[ref36] Li D. Elankumaran P. Kudinha T. Kidsley A. K. Trott D. J. Jarocki V. M.. (2023). Dominance of *Escherichia coli* sequence types ST73, ST95, ST127 and ST131 in Australian urine isolates: a genomic analysis of antimicrobial resistance and virulence linked to F plasmids. Microbial Genomics 9:1–18. doi: 10.1099/mgen.0.001068, PMID: 37471138 PMC10438821

[ref37] Li B. Sun J. Han L. Huang X. Fu Q. Ni Y. (2010). Phylogenetic groups and Pathogenicity Island markers in fecal *Escherichia coli* isolates from asymptomatic humans in China. Appl. Environ. Microbiol. 76, 6698–6700. doi: 10.1128/AEM.00707-10, PMID: 20709835 PMC2950456

[ref38] Manges A. R. Geum H. M. Guo A. Edens T. J. Fibke C. D. Pitout J. D. D. (2019). Global Extraintestinal pathogenic *Escherichia coli* (ExPEC) lineages. Clin. Microbiol. Rev. 32, e00135–e00118. doi: 10.1128/CMR.00135-18, PMID: 31189557 PMC6589867

[ref39] Marin J. Clermont O. Royer G. Mercier-Darty M. Decousser J. W. Tenaillon O.. (2022). The population genomics of increased virulence and antibiotic resistance in human commensal *Escherichia coli* over 30 years in France. Appl. Environ. Microbiol. 88:e0066422. doi: 10.1128/aem.00664-22, PMID: 35862685 PMC9361829

[ref40] Martinson J. N. V. Walk S. T. (2020). *Escherichia coli* residency in the gut of healthy human adults. EcoSal Plus 9:1–19. doi: 10.1128/ecosalplus.esp-0003-2020, PMID: 32978935 PMC7523338

[ref41] Massot M. Daubié A.-S. Clermont O. Jauréguy F. Couffignal C. Dahbi G.. (2016). Phylogenetic, virulence and antibiotic resistance characteristics of commensal strain populations of *Escherichia coli* from community subjects in the Paris area in 2010 and evolution over 30 years. Microbiology 162, 642–650. doi: 10.1099/mic.0.000242, PMID: 26822436 PMC6365622

[ref42] McLellan L. K. Hunstad D. A. (2016). Urinary tract infection: pathogenesis and outlook. Trends Mol. Med. 22, 946–957. doi: 10.1016/j.molmed.2016.09.003, PMID: 27692880 PMC5159206

[ref43] Mobley H. (2016). Measuring *Escherichia coli* gene expression during human urinary tract infections. Pathogens 5:7. doi: 10.3390/pathogens5010007, PMID: 26784237 PMC4810128

[ref44] Moreno E. Andreu A. Pigrau C. Kuskowski M. A. Johnson J. R. Prats G. (2008). Relationship between *Escherichia coli* strains causing acute cystitis in women and the Fecal *E. coli* population of the host. J. Clin. Microbiol. 46, 2529–2534. doi: 10.1128/JCM.00813-08, PMID: 18495863 PMC2519474

[ref45] Moreno E. Johnson J. R. Pérez T. Prats G. Kuskowski M. A. Andreu A. (2009). Structure and urovirulence characteristics of the fecal *Escherichia coli* population among healthy women. Microbes Infect. 11, 274–280. doi: 10.1016/j.micinf.2008.12.002, PMID: 19110067

[ref46] Murase K. Ooka T. Iguchi A. Ogura Y. Nakayama K. Asadulghani M.. (2012). Haemolysin E- and enterohaemolysin-derived haemolytic activity of O55/O157 strains and other *Escherichia coli* lineages. Microbiology 158, 746–758. doi: 10.1099/mic.0.054775-0, PMID: 22194351

[ref47] Naves P. Del Prado G. Huelves L. Gracia M. Ruiz V. Blanco J.. (2008). Correlation between virulence factors and in vitro biofilm formation by *Escherichia coli* strains. Microb. Pathog. 45, 86–91. doi: 10.1016/j.micpath.2008.03.003, PMID: 18486439

[ref48] Nowrouzian F. Adlerberth I. Wold A. E. (2001a). P fimbriae, capsule and aerobactin characterize colonic resident *Escherichia coli*. Epidemiol. Infect. 126, 11–18. doi: 10.1017/S0950268801005118, PMID: 11293669 PMC2869660

[ref49] Nowrouzian F. L. Adlerberth I. Wold A. E. (2006). Enhanced persistence in the colonic microbiota of *Escherichia coli* strains belonging to phylogenetic group B2: role of virulence factors and adherence to colonic cells. Microbes Infect. 8, 834–840. doi: 10.1016/j.micinf.2005.10.011, PMID: 16483819

[ref50] Nowrouzian F. L. Friman V. Adlerberth I. Wold A. E. (2007). Reduced phase switch capacity and functional Adhesin expression of type 1-Fimbriated *Escherichia coli* from immunoglobulin A-deficient individuals. Infect. Immun. 75, 932–940. doi: 10.1128/IAI.00736-06, PMID: 17101646 PMC1828477

[ref51] Nowrouzian F. Hesselmar B. Saalman R. Strannegård I.-L. Åberg N. Wold A. E.. (2003). *Escherichia coli* in infants’ intestinal microflora: colonization rate, strain turnover, and virulence gene carriage. Pediatr. Res. 54, 8–14. doi: 10.1203/01.PDR.0000069843.20655.EE, PMID: 12700366

[ref52] Nowrouzian F. Wold A. E. Adlerberth I. (2001b). P fimbriae and aerobactin as intestinal colonization factors for *Escherichia coli* in Pakistani infants. Epidemiol. Infect. 126, 19–23. doi: 10.1017/S095026880100512X, PMID: 11293677 PMC2869668

[ref53] Östblom A. Adlerberth I. Wold A. E. Nowrouzian F. L. (2011). Pathogenicity Island markers, virulence determinants *malX* and *usp*, and the capacity of *Escherichia coli* to persist in infants’ commensal microbiotas. Appl. Environ. Microbiol. 77, 2303–2308. doi: 10.1128/AEM.02405-10, PMID: 21317254 PMC3067437

[ref54] Paramita R. I. Nelwan E. J. Fadilah F. Renesteen E. Puspandari N. Erlina L. (2020). Genome-based characterization of *Escherichia coli* causing bloodstream infection through next-generation sequencing. PLoS One 15:e0244358. doi: 10.1371/journal.pone.0244358, PMID: 33362261 PMC7757869

[ref55] Pokharel P. Dhakal S. Dozois C. M. (2023). The diversity of *Escherichia coli* Pathotypes and vaccination strategies against this versatile bacterial pathogen. Microorganisms 11:344. doi: 10.3390/microorganisms11020344, PMID: 36838308 PMC9965155

[ref56] Poolman J. T. Wacker M. (2016). Extraintestinal pathogenic *Escherichia coli*, a common human pathogen: challenges for vaccine development and Progress in the field. J. Infect. Dis. 213, 6–13. doi: 10.1093/infdis/jiv429, PMID: 26333944 PMC4676548

[ref57] Pusz P. Bok E. Mazurek J. Stosik M. Baldy-Chudzik K. (2014). Type 1 fimbriae in commensal *Escherichia coli* derived from healthy humans. Acta Biochim. Pol. 61:389–392. doi: 10.18388/abp.2014_1912, PMID: 24851235

[ref58] Raimondi S. Righini L. Candeliere F. Musmeci E. Bonvicini F. Gentilomi G.. (2019). Antibiotic resistance, virulence factors, phenotyping, and genotyping of *E. coli* isolated from the feces of healthy subjects. Microorganisms 7:251. doi: 10.3390/microorganisms7080251, PMID: 31405113 PMC6722543

[ref59] Reisner A. Krogfelt K. A. Klein B. M. Zechner E. L. Molin S. (2006). In vitro biofilm formation of commensal and pathogenic *Escherichia coli* strains: impact of environmental and genetic factors. J. Bacteriol. 188, 3572–3581. doi: 10.1128/JB.188.10.3572-3581.2006, PMID: 16672611 PMC1482849

[ref60] Restieri C. Garriss G. Locas M.-C. Dozois C. M. (2007). Autotransporter-encoding sequences are phylogenetically distributed among *Escherichia coli* clinical isolates and reference strains. Appl. Environ. Microbiol. 73, 1553–1562. doi: 10.1128/AEM.01542-06, PMID: 17220264 PMC1828755

[ref61] Riley L. W. (2014). Pandemic lineages of extraintestinal pathogenic *Escherichia coli*. Clin. Microbiol. Infect. 20, 380–390. doi: 10.1111/1469-0691.12646 24766445

[ref62] Russo T. A. Johnson J. R. (2003). Medical and economic impact of extraintestinal infections due to *Escherichia coli*: focus on an increasingly important endemic problem. Microbes Infect. 5, 449–456. doi: 10.1016/S1286-4579(03)00049-2 12738001

[ref63] Sabaté M. Moreno E. Pérez T. Andreu A. Prats G. (2006). Pathogenicity island markers in commensal and uropathogenic *Escherichia coli* isolates. Clin. Microbiol. Infect. 12, 880–886. doi: 10.1111/j.1469-0691.2006.01461.x, PMID: 16882293

[ref64] Sarowska J. Futoma-Koloch B. Jama-Kmiecik A. Frej-Madrzak M. Ksiazczyk M. Bugla-Ploskonska G.. (2019). Virulence factors, prevalence and potential transmission of extraintestinal pathogenic *Escherichia coli* isolated from different sources: recent reports. Gut Pathog 11:10. doi: 10.1186/s13099-019-0290-0, PMID: 30828388 PMC6383261

[ref65] Schubert S. Rakin A. Heesemann J. (2004). The Yersinia high-pathogenicity island (HPI): evolutionary and functional aspects. Int. J. Med. Microbiol. 294, 83–94. doi: 10.1016/j.ijmm.2004.06.026, PMID: 15493818

[ref66] Schwan W. R. Lee J. L. Lenard F. A. Matthews B. T. Beck M. T. (2002). Osmolarity and pH growth conditions regulate *fim* gene transcription and type 1 pilus expression in Uropathogenic *Escherichia coli*. Infect. Immun. 70, 1391–1402. doi: 10.1128/IAI.70.3.1391-1402.2002, PMID: 11854225 PMC127777

[ref67] Scribano D. Sarshar M. Prezioso C. Lucarelli M. Angeloni A. Zagaglia C.. (2020). D-mannose treatment neither affects Uropathogenic *Escherichia coli* properties nor induces stable FimH modifications. Molecules 25:316. doi: 10.3390/molecules25020316, PMID: 31941080 PMC7024335

[ref68] Searle L. J. Méric G. Porcelli I. Sheppard S. K. Lucchini S. (2015). Variation in Siderophore biosynthetic gene distribution and production across environmental and Faecal populations of *Escherichia coli*. PLoS One 10:e0117906. doi: 10.1371/journal.pone.0117906, PMID: 25756870 PMC4355413

[ref69] Shah C. Baral R. Bartaula B. Shrestha L. B. (2019). Virulence factors of uropathogenic *Escherichia coli* (UPEC) and correlation with antimicrobial resistance. BMC Microbiol. 19:204. doi: 10.1186/s12866-019-1587-3, PMID: 31477018 PMC6720075

[ref70] Shruthi N. (2012). Phenotypic study of virulence factors in *Escherichia Coli* isolated from antenatal cases, catheterized patients, and Faecal Flora. JCDR 6, 1699–1703. doi: 10.7860/JCDR/2012/4669.2634, PMID: 23373032 PMC3552208

[ref71] Sintsova A. Frick-Cheng A. E. Smith S. Pirani A. Subashchandrabose S. Snitkin E. S.. (2019). Genetically diverse uropathogenic *Escherichia coli* adopt a common transcriptional program in patients with UTIs. eLife 8:e49748. doi: 10.7554/eLife.49748, PMID: 31633483 PMC6802966

[ref72] Sobieszczańska B. M. (2007). Hemolizyny *Escherichia coli*. Post Mikrobiol 46, 343–353.

[ref73] Starčič Erjavec M. Žgur-Bertok D. (2015). Virulence potential for extraintestinal infections among commensal *Escherichia coli* isolated from healthy humans—the Trojan horse within our gut. FEMS Microbiol. Lett. 362:1–9. doi: 10.1093/femsle/fnu061, PMID: 25657191

[ref74] Subashchandrabose S. Hazen T. H. Brumbaugh A. R. Himpsl S. D. Smith S. N. Ernst R. D.. (2014). Host-specific induction of *Escherichia coli* fitness genes during human urinary tract infection. Proc. Natl. Acad. Sci. USA 111, 18327–18332. doi: 10.1073/pnas.1415959112, PMID: 25489107 PMC4280598

[ref75] Tenaillon O. Skurnik D. Picard B. Denamur E. (2010). The population genetics of commensal *Escherichia coli*. Nat. Rev. Microbiol. 8, 207–217. doi: 10.1038/nrmicro2298, PMID: 20157339

[ref76] The R Core Team (2024). R: A language and environment for statistical computing. R Core Team Technical Report R Foundation for Statistical Computing, Vienna, Austria.

[ref77] Vollmerhausen T. L. Ramos N. L. Gündoğdu A. Robinson W. Brauner A. Katouli M. (2011). Population structure and uropathogenic virulence-associated genes of faecal *Escherichia coli* from healthy young and elderly adults. J. Med. Microbiol. 60, 574–581. doi: 10.1099/jmm.0.027037-0, PMID: 21292854

[ref78] Weissman S. J. Hansen N. I. Zaterka-Baxter K. Higgins R. D. Stoll B. J. (2016). Emergence of antibiotic resistance-associated clones among *Escherichia coli* recovered from newborns with early-onset Sepsis and meningitis in the United States, 2008–2009: table 1. J Ped Infect Dis 5, 269–276. doi: 10.1093/jpids/piv013, PMID: 26407251 PMC5125450

[ref79] Wirth T. Falush D. Lan R. Colles F. Mensa P. Wieler L. H.. (2006). Sex and virulence in *Escherichia coli*: an evolutionary perspective. Mol. Microbiol. 60, 1136–1151. doi: 10.1111/j.1365-2958.2006.05172.x, PMID: 16689791 PMC1557465

