# Supplementary material for: Extraintestinal traits of pathogenicity and sequence type lineages in commensal Escherichia coli from adults and young children: genotypic and phenotypic profiles
Source: Front Microbiol. 2025 May 26;16:1579685. doi: 10.3389/fmicb.2025.1579685 (PMC12146316; doi:10.3389/fmicb.2025.1579685)
Supplement: Supplementary file 1 [file Table_1.DOCX]

**Supplementary Table S1. Hemolytic activity evaluation among *E. coli* isolates, positive for *hlyA* gene, from adults and young children.**

| **Strain** | **Hemolytic zone (mm)** | **Hemolytic activity** |
| --- | --- | --- |
| **Adults** |  |  |
| 4 | 1.5 | ++ |
| 5 | 2.5 | ++ |
| 6 | 1.5 | ++ |
| 9 | 2 | ++ |
| 11 | 1.5 | ++ |
| 16 | 3 | ++ |
| 24 | 2 | ++ |
| 34 | 1.5 | ++ |
| 44 | 2.5 | ++ |
| 52 | 2 | ++ |
| 60 | 3 | ++ |
| 70 | 1 | ++ |
| 71 | 2 | ++ |
| 72 | - | - |
| 83 | 3 | ++ |
| 85 | 2 | ++ |
| 91 | 2 | ++ |
| 98 | 2.5 | ++ |
| 113 | 3 | ++ |
| 116 | 1.5 | ++ |
| 120 | 1.5 | ++ |
| 123 | 1 | ++ |
| 127 | 2 | ++ |
| 130 | 2 | ++ |
| 131 | 1.5 | ++ |
| 133 | 2 | ++ |
| 135 | 3 | ++ |
| 138 | 2.5 | ++ |
| 139 | 1.5 | ++ |
| 141 | 1 | ++ |
| **Young children** |  |  |
| 4 | 3 | ++ |
| 13 | - | - |
| 22 | 3 | ++ |
| **Positive control**  ATCC 35218 | 2 | ++ |

The haemolytic activity was graded as follows: -, no haemolysis; +, less than 1 mm; ++, 1–5 mm; and +++, more than 5 mm.
