# Supplementary material for: Extraintestinal traits of pathogenicity and sequence type lineages in commensal Escherichia coli from adults and young children: genotypic and phenotypic profiles
Source: Front Microbiol. 2025 May 26;16:1579685. doi: 10.3389/fmicb.2025.1579685 (PMC12146316; doi:10.3389/fmicb.2025.1579685)
Supplement: Supplementary file 2 [file Table_2.DOCX]

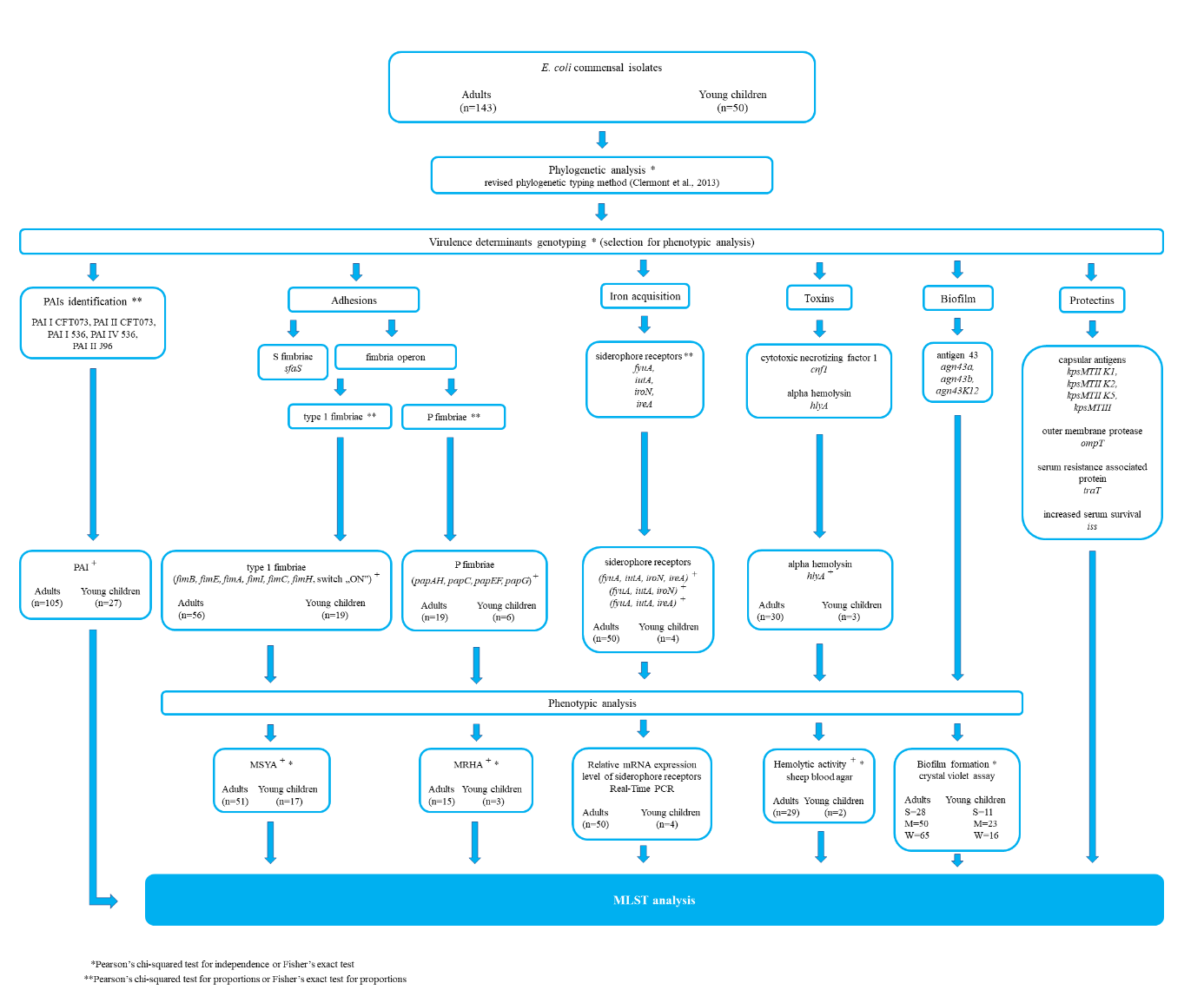


**Supplementary Figure S1. Experimental design**


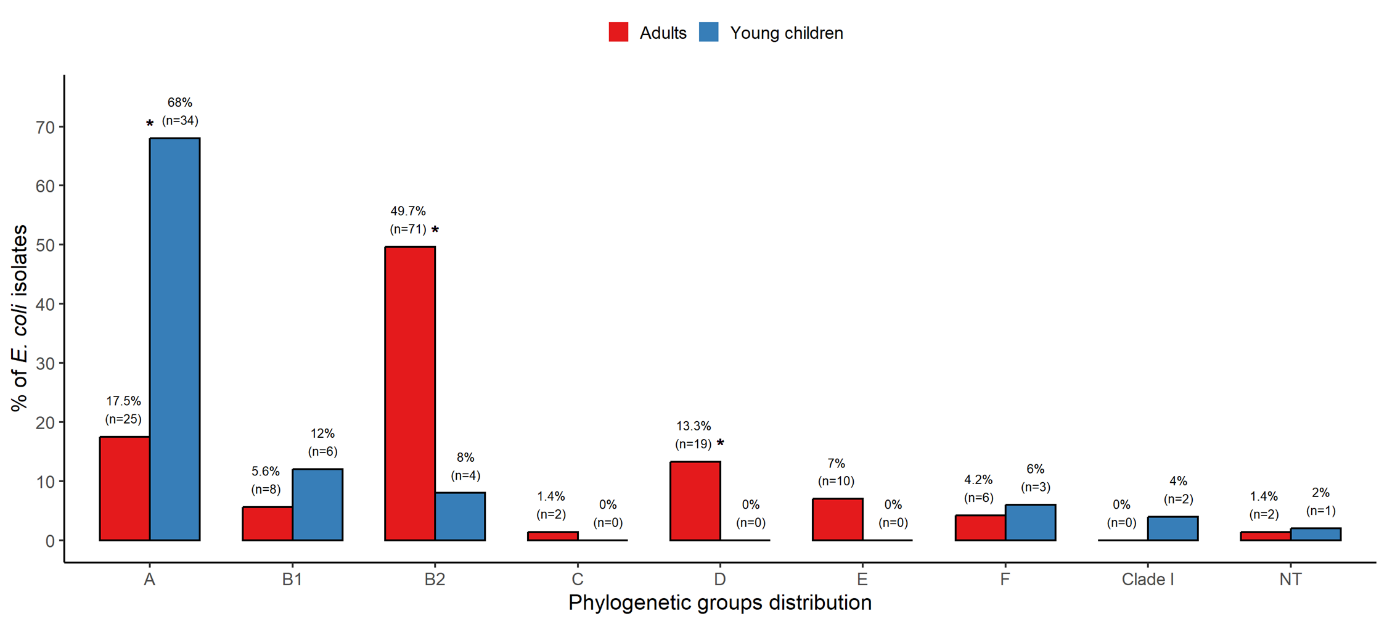


**Supplementary Figure S2.** **Extended phylogenetic structure of *E. coli* isolates derived from adults and young children.** NT— not typeable, * - statistically significant


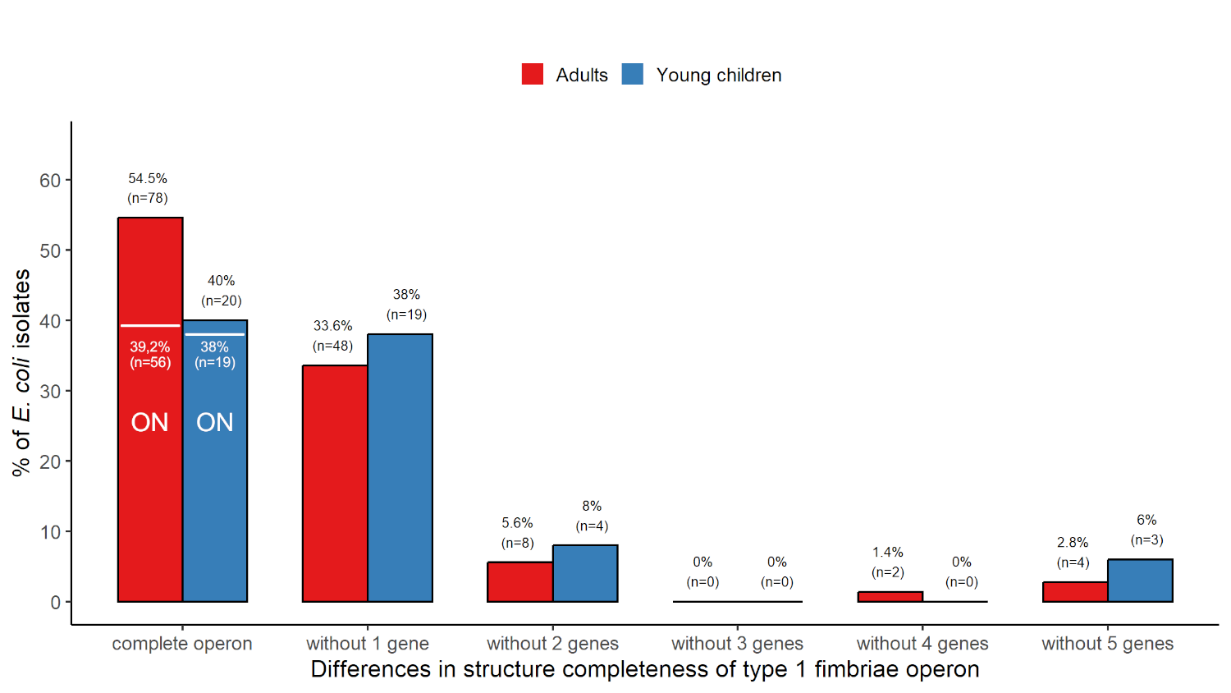


**Supplementary Figure S3. Frequency of the gene number combinations within the structure of type 1 fimbria operon among the *E. coli* isolates from adults and young children.**


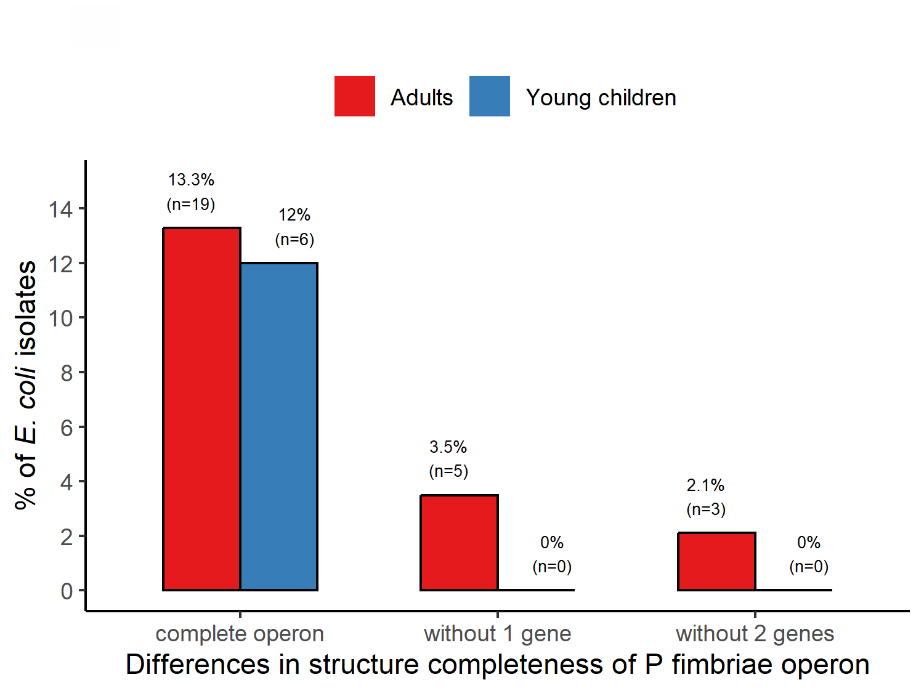


**Supplementary Figure S4. Frequency of the gene number combinations within the structure of P fimbria operon among the *E. coli* isolates from adults and young children.**








**Supplementary Figure S5.** **The analysis of biofilm formation ability among *E. coli* isolates from adults (A) and young children (B).**


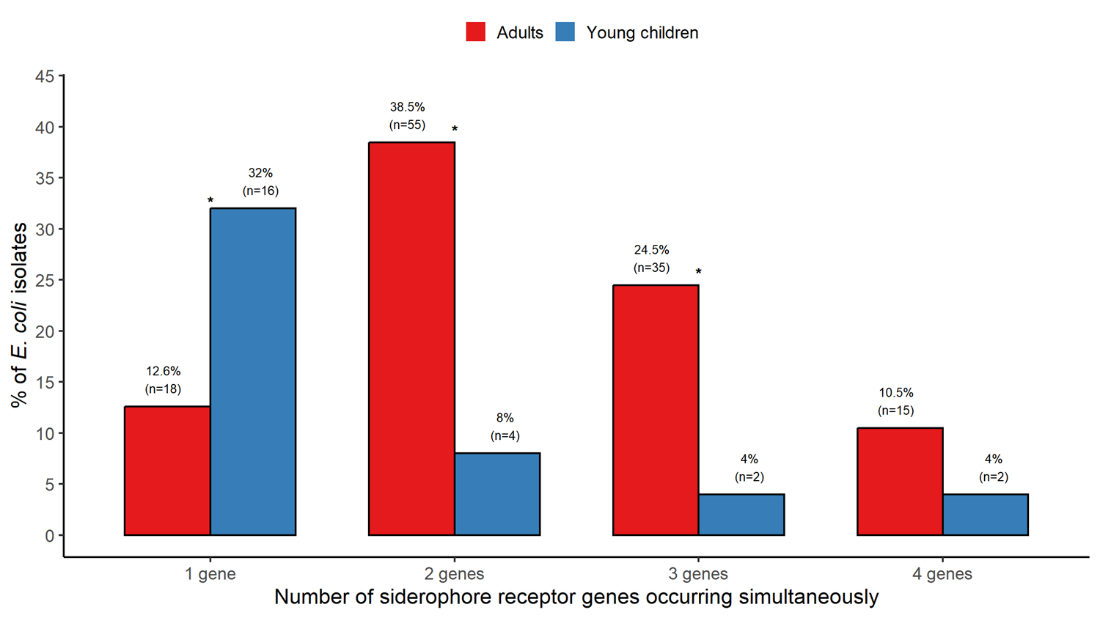


**Supplementary Figure S6. Analysis of the simultaneous prevalence of siderophore receptor genes among *E. coli* isolates from adults and young children.** * - statistically significant
