# Supplementary figures and images for: Extraintestinal traits of pathogenicity and sequence type lineages in commensal Escherichia coli from adults and young children: genotypic and phenotypic profiles
Source: Front Microbiol. 2025 May 26;16:1579685. doi: 10.3389/fmicb.2025.1579685 (PMC12146316; doi:10.3389/fmicb.2025.1579685)

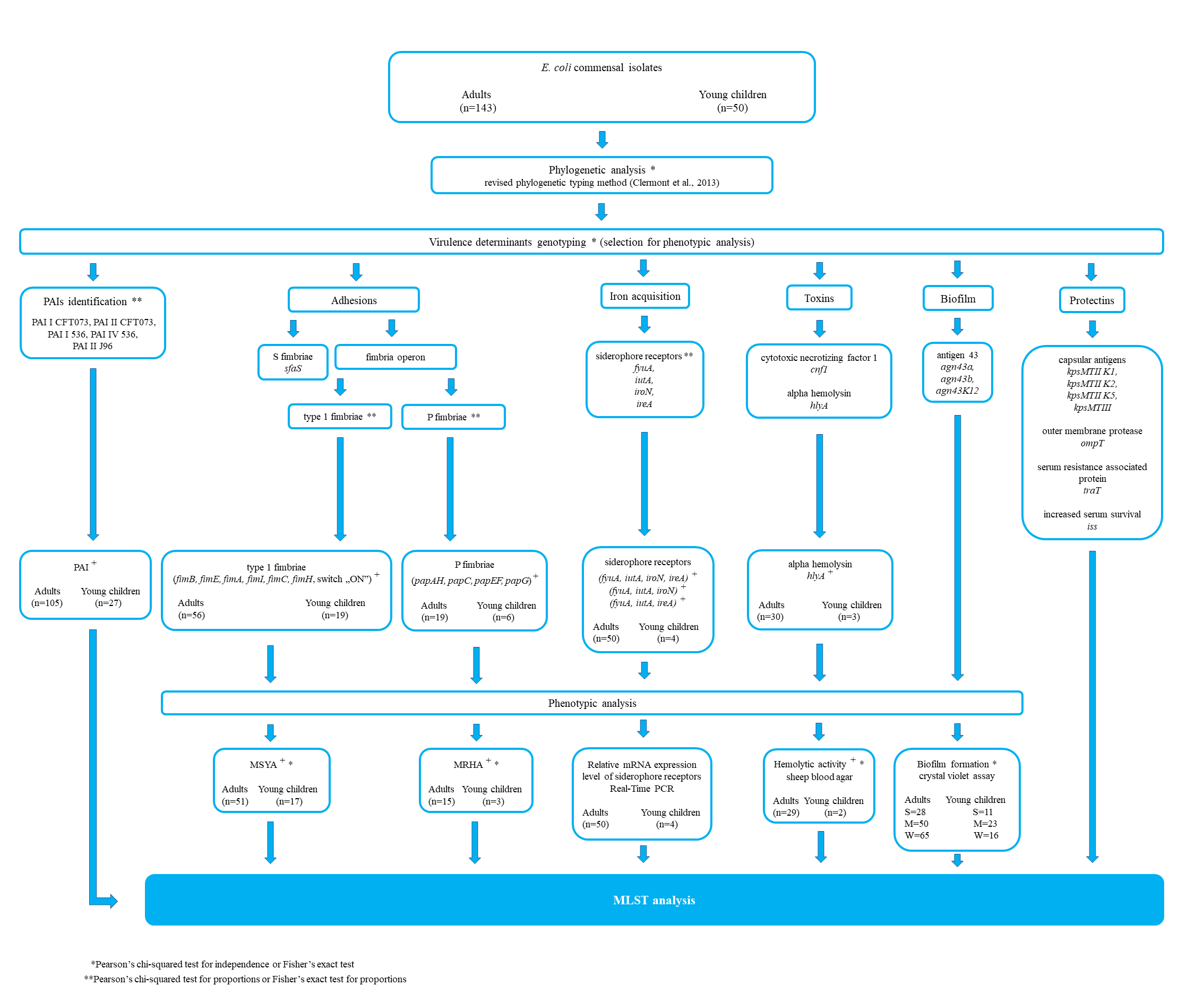

Supplement: Supplementary file 3 [file Image_1.TIF]

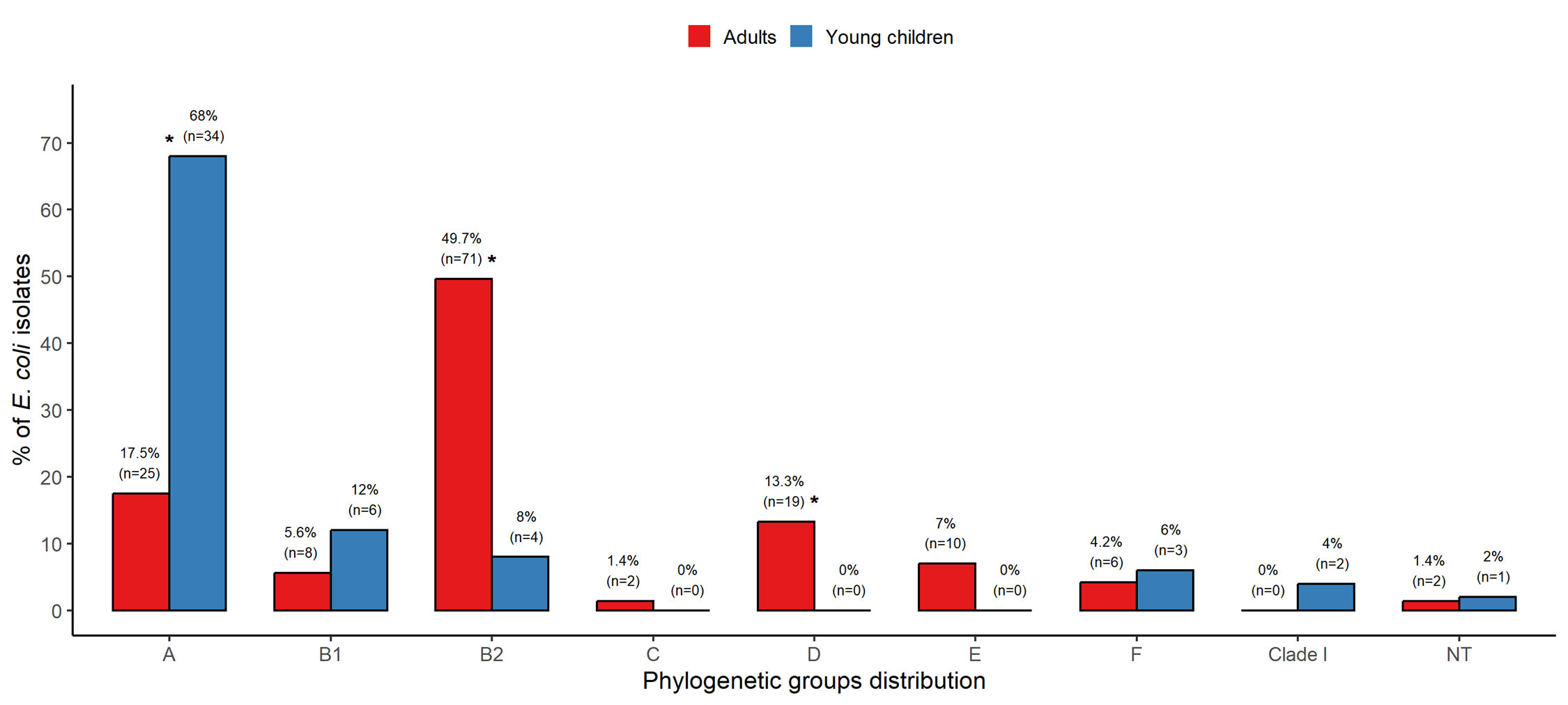

Supplement: Supplementary file 4 [file Image_2.TIF]

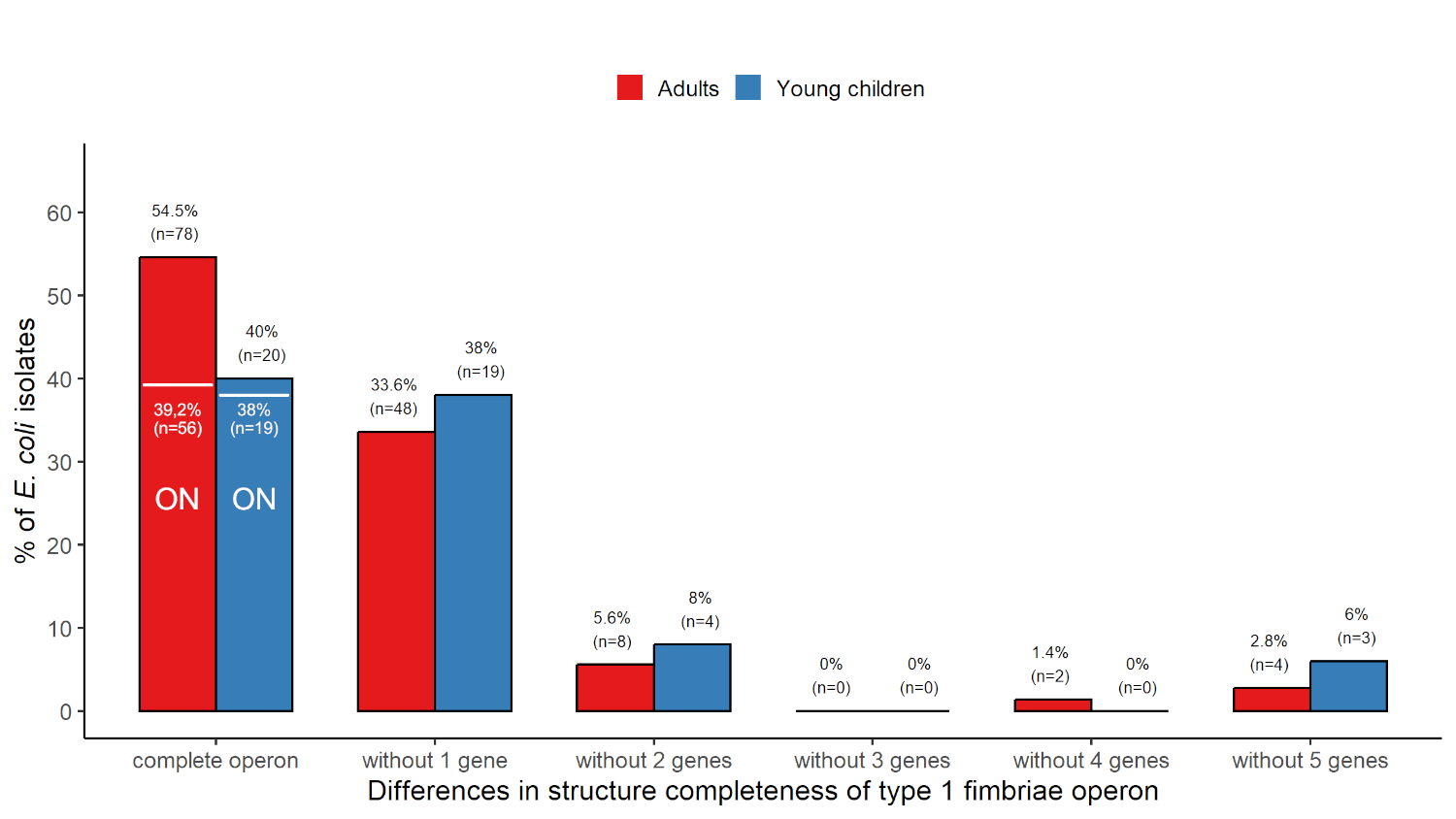

Supplement: Supplementary file 5 [file Image_3.TIF]

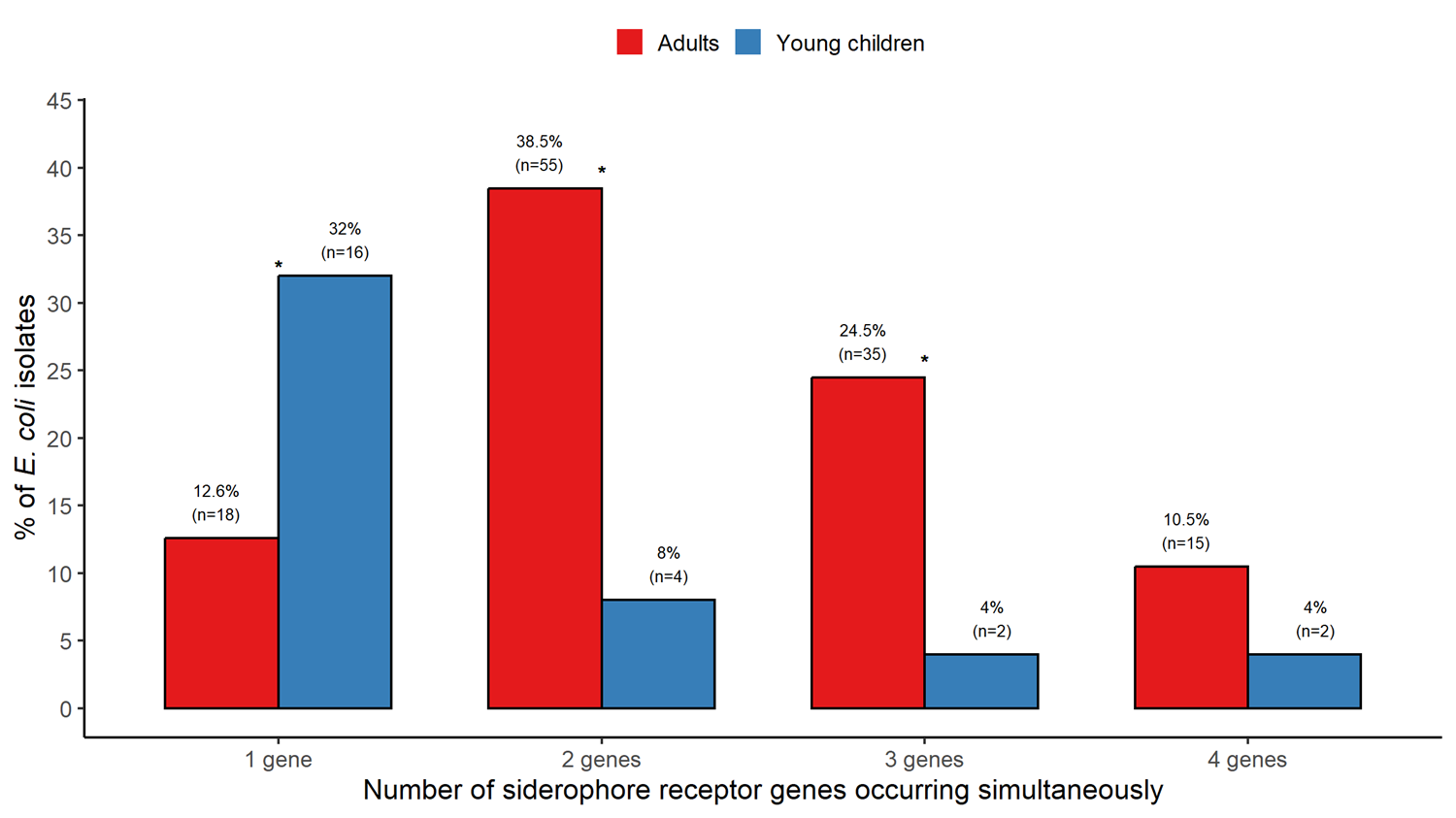

Supplement: Supplementary file 8 [file Image_6.TIF]

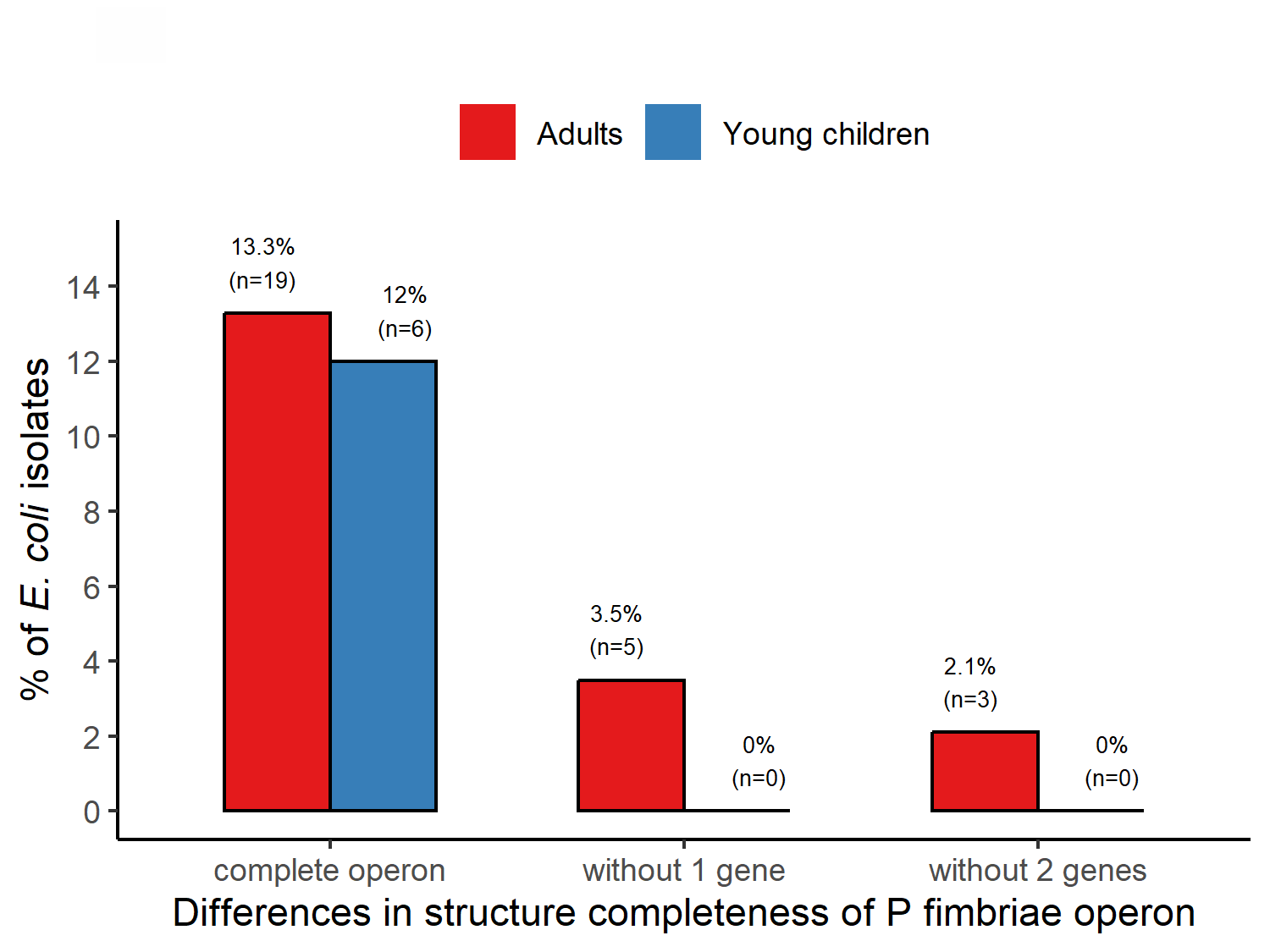

Supplement: Supplementary file 9 [file Image_7.TIF]
